# How knee muscles and ground reaction forces shape knee buckling and ankle push-off in neuromuscular simulations of human walking

**DOI:** 10.1038/s41598-025-86147-z

**Published:** 2025-01-17

**Authors:** Alexandra Buchmann, Bernadett Kiss, Alexander Badri-Spröwitz, Daniel Renjewski

**Affiliations:** 1 Chair of Applied Mechanics, Technical University of Munich, Garching, 85748 Germany; 2 Dynamic Locomotion Group, Max-Plank-Institute for Intelligent Systems, Stuttgart, 70569 Germany; 3 Department of Mechanical Engineering, KU Leuven, Leuven, 3001 Belgium

**Keywords:** Ankle push-off, Knee, Gastrocnemius, Hamstrings, Vastus, Predictive neuromuscular simulation, Computational models, Motor control, Bone quality and biomechanics, Musculoskeletal models

## Abstract

Ankle push-off is important for efficient, human-like walking, and many prosthetic devices mimic push-off using motors or elastic elements. The knee is extended throughout the stance phase and begins to buckle just before push-off, with timing being crucial. However, the exact mechanisms behind this buckling are still unclear. We use a predictive neuromuscular simulation to investigate whether active muscles are required for knee buckling and to what extent ground reaction forces (GRFs) drive it. In a systematic parameter search, we tested how long the knee muscles vastus (VAS), gastrocnemius (GAS), and hamstrings could be deactivated while maintaining a stable gait with impulsive push-off. VAS deactivation up to 35% of the gait cycle resulted in a dynamic gait with increased ankle peak power. GAS deactivation up to 20% of the gait cycle was detrimental to gait efficiency and showed reduced ankle peak power. At the start of knee buckling, the GRF vector is positioned near the knee joint’s neutral axis, assisting in knee flexion. However, this mechanism is likely not enough to drive knee flexion independently. Our findings contribute to the biomechanical understanding of ankle push-off, with applications in prosthetic and bipedal robotic design, and fundamental research on human gait mechanics.

## Introduction

Healthy neuromechanics are essential for efficient, robust, and agile human walking. When key components like muscles, tendons, and the neural control system of the legs degrade or do not develop properly, our mobility is significantly reduced. Severe cases include conditions like cerebral palsy, which can cause limping, toe-walking, and flat feet^1^, or the effects of aging^2,3^. The ankle push-off is critical for efficient and robust walking as it shapes the natural leg dynamics^4–6^. It depends on all leg components working together, highlighting the need for fundamental scientific research into the catapult mechanism, its parts, and how they interact.

The ankle push-off is an impulsive power burst generated at the ankle joint at the end of stance^7^. Hof et al.^8^ identified the push-off as a catapult action. A catapult needs three functional elements: an elastic element to store energy, a block to counteract the reaction forces generated during loading and release, and a catch mechanism for slow energy storage and precise timing of rapid energy release to accelerate the projectile.

For the ankle push-off in human gait, the foot in contact with the ground blocks the ankle motion and counters the ground reaction forces (GRFs) during stance^7^. The Achilles tendon is the elastic element that stores energy during stance^9^ and rapidly recoils at push-off^10–12^. The muscles attached to the Achilles tendon, the gastrocnemius (GAS) and soleus (SOL), work isometrically during stance^11,12^, which is an energetically efficient mode of muscle operation. The rapid recoil results in the impulsive power output at the ankle joint, preparing the trailing leg for^13^ and accelerating it^7^ into swing. The positive magnitude of the ankle power during push-off is higher than the peak power used to elastically load the tendon, representing a power amplification^7,9^. Simulation^14^ and robotic hardware^15^ have shown that passive elastic structures inducing torque at the ankle are sufficient to generate steady-state walking featuring an impulsive ankle push-off. Overall, the block and elastic elements of the human swinging leg catapult are well understood. However, the catch mechanism with the functional elements that facilitate the locking and releasing of the catapult, still needs to be identified and described in detail in the context of human gait.

**Fig. 1:**
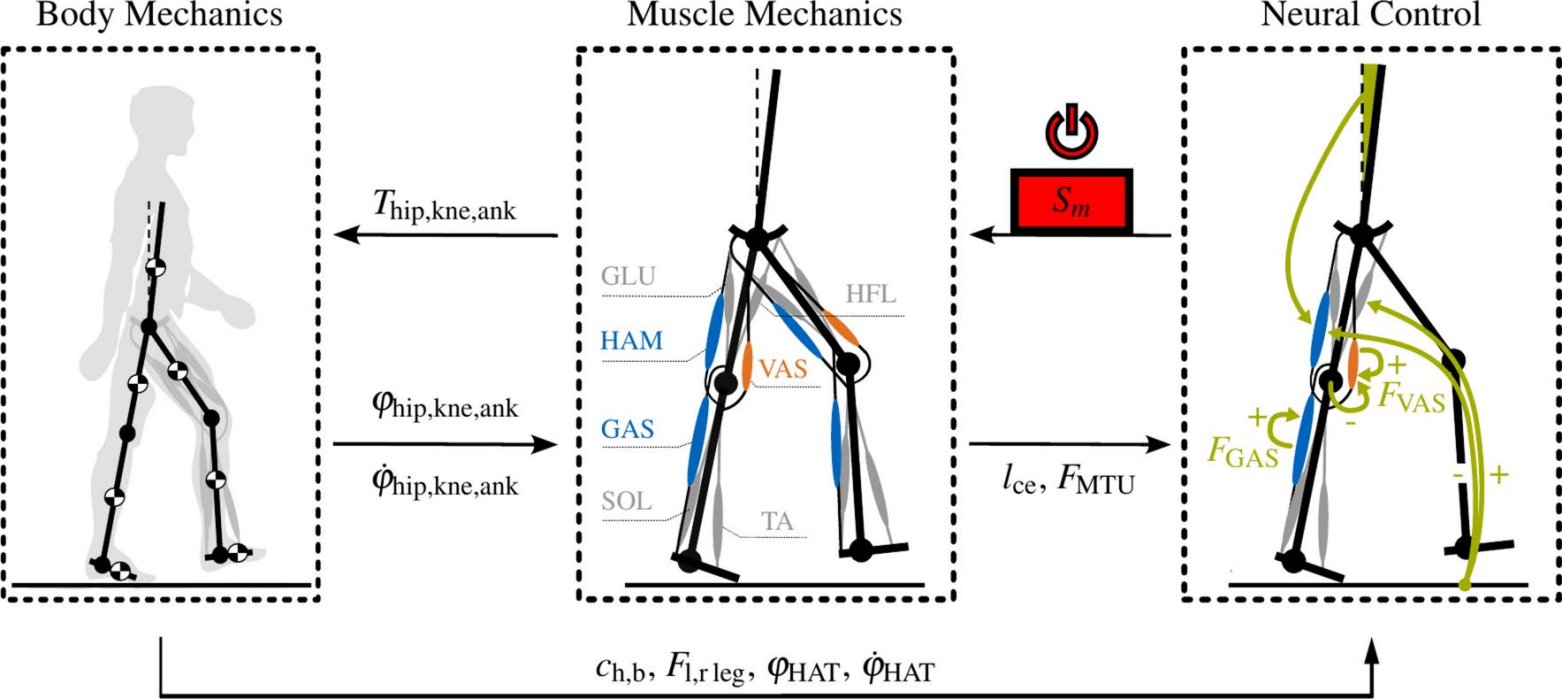
Setup and signal flow in the pNMS^29^. The multi-body simulation has nine degrees of freedom. Revolute joints in the hips, knees, and ankles connect the trunk, femur, tibia, and foot. Based on joint angles $\varphi$ and angular velocities $\dot{\varphi }$, the muscle model calculates the current length and force for each of the seven muscles per leg. A neural feedback controller computes the individual muscle stimulations $S_m$ based on the current muscle length $l_\text {ce}$, the MTU force $F_\text {MTU}$, the head-arm-trunk segment (HAT) angle and angular velocity $\varphi _\text {HAT}$ and $\dot{\varphi }_\text {HAT}$, the ground contact of heel and ball $c_\text {h,b}$, and the body load on the left and right leg $F_\text {l,r leg}$. The muscle-tendon forces resulting from the given muscle stimulation and the passive, elastic tendon properties are multiplied by their respective lever arms and summed for all muscles. The total joint torques *T* are fed back into the multi-body simulation. We focus on the VAS, GAS, and HAM and deactivate them for defined periods.

The timing of ankle push-off is critical for energy-efficient gait^16^. The knee, which has been extended throughout stance, buckles at 64% of stance^7^ before push-off starts. Push-off begins when the ankle power becomes positive with the alleviation phase at 78% of stance^7^. At this time, the ankle joint also begins to extend. At 82% of stance^7^, the contralateral leg touches down. The GRF vector that passed in front of the knee during single support passes behind the knee joint with the contra-lateral leg’s touch-down (TDc), see 82% stance figure in Ref.^7^, Fig. 1 and^13^, Fig. 5-14 and 5-15, or as stated in Ref.^13^, the knee moves in front of the vector. With the load transfer and the shift of the center of pressure to the forefoot, the tibia rotates, and the ankle rapidly plantarflexes^13^ with the recoil of the Achilles tendon and impulsive power release. At 83% stance, the beginning of preswing, the hip begins to flex, which Perry et al.^13^ describe as *“a reaction”* to the ankle mechanics and load transfer to the opposite leg. The hip flexor muscles (HFL) are activated just before preswing until midway through leg swing^13^. Dietz and Harkema^17^ and Pang and Yang^17,18^ found evidence from in vivo experiments that sensory information, specifically afferent input from hip joint extension and load receptors, may play a critical role in generating locomotor patterns in the human spinal cord.

The precise timing of the push-off release described above requires accurate segment and joint synchronization. Biarticular muscle-tendon units (MTUs) that span two joints are known for their role in joint synchronization^19^ with defined moment arm ratios and long tendons to support recoil and the use of elastic energy^20^. In humans, the GAS spans the knee and ankle joints, and hamstrings (HAM) spans the knee and hip joints. In several studies of lower limb prostheses, including the GAS has been shown to contribute to energy efficiency by increasing ankle push-off power^21–23^. While the overall sequence of events around push-off is established, it is not yet fully understood how ankle mechanics, knee and hip flexion, and load transfer lead to one another.

In contrast to humans, catapult mechanisms and their triggering mechanisms are well understood in several animal species. Gronenberg^24^ categorized three catapult release mechanisms in small animals: (1) passive deformation mechanisms, (2) active latching with trigger muscles, and (3) triggers based on antagonistic coactivation. In larger animals, (4) GRFs have been found to play a central role in counteracting tendon forces instead of antagonistic muscles^25^. The functions of the catapult mechanisms in animals offer potential insights that may enhance our understanding of human biomechanics and are therefore briefly discussed below. (1) Passive mechanisms rely on the passive deformation of a latch. The latch deforms slowly, and when the force is great enough, the latch buckles and releases the catapult. Springtails, mites, and flea beetles use passive deformation mechanisms for jumping^24^. (2) Active latching with trigger muscles is used when precise, deliberate timing is needed. Examples are the trap-jaw ant^26^, flea jumping^27^, or jaw depression during suction feeding of fish^28^. Large, powerful muscles provide the energy to stretch tendons and store elastic energy. The joints and bone segments, where the MTUs are attached, are designed to remain in an elastically loaded equilibrium position until a trigger muscle disturbs the equilibrium to release the catapult. (3) The coactivation of strong antagonistic muscles can also facilitate active catapult mechanisms^24^. The strong antagonist muscle counteracts the forces of the elastically loaded MTU to keep the system at rest. To release the catapult, the antagonist quickly relaxes. (4) Wilson et al.^25^ identified a “geometric” catapult release for rapid limb extension in galloping horses that reduces the need for active muscle work to actuate the swing leg. Throughout stance, the GRF vector passes anteriorly of the elbow and carpus joints, keeping the leg straight. The MTUs of the biceps and digital flexors resist the joint motion and store elastic energy, similar to the GAS and SOL MTUs in the human calf. At the end of stance, the trunk passes in front of the GRF vector. The new relative orientation of the trunk and the GRF reverses the direction of joint loading, allowing the carpus joint to buckle, slackening the limb. Without the GRF acting on the limb, the stretched MTUs quickly recoil, flexing the elbow, extending the shoulder joint, and swinging the leg forward.

As described previously, knee buckling is needed in human walking prior to ankle push-off^7^. Therefore, this paper will examine the role of the knee muscles in the ankle push-off process. We use a predictive neuromuscular simulation (pNMS) with a local reflex controller^29^ to investigate two research questions. (I) Are active muscles required for knee buckling in late stance for push-off initiation, and how do the biarticular muscles GAS and HAM influence the buckling? Second, we investigate whether a similar geometric catapult mechanism described in horses^25^ may also play a role in human gait: (II) To what extent can GRFs drive knee buckling in late stance? With a systematic search, we tested how long and when in the gait cycle the knee muscles vastus group (VAS), GAS, and HAM could be individually deactivated while maintaining a stable, dynamic gait. We evaluated how the gait changed without the joint torque contributions from the selected muscles. The analysis focused on the time around push-off, 50-62% of the gait cycle. The muscle deactivation allows us to evaluate individual contributions to knee buckling and push-off release, an approach not feasible in human experiments.

## Methods

### Predictive neuromuscular simulation

 For our study, we simulate the human gait with a 2D reflex-controlled pNMS with default control parameters from^29^ running in Matlab Simulink R2023a (The MathWorks Inc., Natick, Massachusetts, USA). The same control parameters were applied in all deactivation experiments, i.e, without reoptimizing the controller. The model features and structures relevant to this work are summarized below. A complete description of the model can be found in^29^.

Figure 1 illustrates the simulation structure and highlights the muscle models and selected reflexes. The multi-body simulation has nine degrees of freedom (planar trunk-to-world three; knees, ankles and hips - one each for both legs) and consists of seven rigid bodies, including a trunk and the femur, tibia, and foot of each leg, connected by revolute joints. Seven Hill-type muscle models per leg actuate the bodies, including the GAS, SOL, VAS, tibialis anterior (TA), gluteus group (GLU), HFL, and HAM. In Fig. 1, the muscles studied in the paper are highlighted in orange for monoarticular muscles and in blue for biarticular muscles. All other muscles that remain unchanged are shown in gray.

Decentralized reflex feedback controllers coordinate muscle movements by computing the muscle stimulus $S_m(t)$ of a given muscle *m* based on low-level sensory information such as muscle force, length, torso orientation, and ground contact^29^, i.e., whether the respective leg is in the stance or swing phase. Figure 1 shows the stance reflexes for the VAS, GAS, and HAM. The GAS and VAS are controlled by positive force feedback, where

1 $$\begin{aligned} S_m(t) = S_{0,m} + G_m F_m (t - \Delta t_m) \text {\hspace{0.25cm} with \hspace{0.25cm}} \tau \frac{d a_m(t)}{dt} = S_m(t) - a_m(t) \end{aligned}$$

with a resting stimulation $S_{0,m}$, a muscle-specific reflex time delay $\Delta t_m$, the muscle force $F_m(t)$, and the reflex feedback gain $G_m$.

From the stimulation, the muscle activation $a_m$ is calculated via the excitation-contraction coupling^30^ with the time constant $\tau$.

The VAS is inhibited if the knee is overextended beyond 175 deg. The HAM is activated based on the upper body tilt angle for postural control and the weight percentage carried by the respective stance leg. In addition, the touchdown of the contralateral leg modulates the muscle activations of HFL and HAM. HFL is activated, and HAM is inhibited, as indicated by the plus and minus signs in Fig. 1.

### Experimental setup

 We implemented a custom Simulink library module to deactivate the stimulation of a specific muscle $S_m(t \in \Delta t^*) = 0$ for a defined time period $\Delta t^*$. The stimulation activates the contractile element of the Hill-type muscle model^30^. The contractile and series elastic element are modeled together, while the latter features the passive elastic properties of the MTU’s tendon. When the stimulation is removed, the contractile element cannot exert any forces; therefore, the serial elastic element also cannot generate any forces. For numerical stability, our model has a so-called parallel elastic element and a buffer elasticity, both in parallel to the contractile element^29^. The buffer and parallel elasticity are only relevant outside the nominal working range of the MTU and play a minor role in the locomotion dynamics^29^.

We evaluated three experiments with individual deactivation of the GAS, HAM, and VAS. The remaining muscles, marked in gray in Fig. 1, were kept intact according to the original model configuration. The torques induced by the joint limits in the knee, hip, and ankle as described in^29^, p. 271, Appendix IV also remained. The muscle stimulation was switched off at a defined point in the gait cycle ($\text {gc}_\text {off} \in [0,100]\%$) and was kept switched off up to 1 s ($t_\text {off} \in [0,1]\,s$). The average stride time of the reference simulation is 1.25 s (table 1). Removing any muscle stimulation for more than 80%, i.e., 1 s, of the stride led to immediate failure in preliminary experiments, so a maximum duration of 1 s was sufficient to capture the entire solution space. The pNMS needs several strides to settle into steady walking. We, therefore, waited six strides before turning off the muscle stimulation. Muscle deactivation was applied to both legs, with each limb deactivated at the corresponding gait event, so that, for example, heel-off (HO) of the left leg will trigger muscle deactivation in the left leg. A timer started when the respective leg touched the ground. When the timer reached $\text {gc}_\text {off} \cdot t_\text {stride}$, the selected muscle stimulation was set to zero. The stride time $t_\text {stride}$ was estimated online based on the previous stride. We simulated all combinations of $\text {gc}_\text {off}$ between 0% and 100% stride with 50 equal increments (2% length) and $t_\text {off}$ between 0 and 1 s with 400 equal increments (0.025 s long). The simulations ran for 20 s total simulation time to ensure that the model is able to reach steady state walking with muscles turned off after the sixth step. The simulation was carried out in parallel on 16 cores on a Windows machine with an Intel Xeon E5-2660v4 @ 2.0 GHz and 64GB RAM. Each pNMS was solved using Matlab *ode23tb*, a variable step size solver for stiff differential equations using the trapezoidal rule and backward differentiation^31,32^. We applied the automatic solver settings of Matlab: a maximum solver step size of 0.1 s, a minimum step size of $1\times 10^{-11}$ s, a relative tolerance of $1\times 10^{-3}$, and disabled zero crossing detection for the ground contact model.

### Data processing

 First, we computed a stability score as the percentage [%] of the total desired simulation time:

2 $$\begin{aligned} \text {Score} = \frac{t_\text {end} - t_\text {stride\,6}}{t_\text {sim, des} - t_\text {stride\,6}} \cdot 100, \end{aligned}$$

where $t_\text {end}$ is the end time for each simulation, $t_\text {sim, des} = 20\,s$ is the desired total simulation time, and $t_\text {step\,6}$ is the simulation time until stride six when we started the muscle deactivation.

Five measures characterizing the ankle push-off were analyzed to assess the individual muscle contributions to push-off and the timing of energy release. Only simulations that walked 20 s without failure were considered for further analysis. The measures are motivated by^7^. The ankle power amplification is defined as the ratio of the maximum negative ankle power $P_\text {min, ank}$ to the maximum positive power $P_\text {max, ank}$ during stance with

3 $$\begin{aligned} P_\text {amp, ank} = \left| \frac{P_\text {max, ank}}{P_\text {min, ank}} \right| . \end{aligned}$$

The trailing leg’s momentum change helps to assess how effectively the leg is accelerated into swing during push-off. We computed the momentum change from the beginning of the alleviation phase $t_\text {a}$ to toe-off (TO) $t_\text {to}$. The alleviation phase marks the beginning of the push-off defined by the start of positive power output at the ankle joint^7^. The momentum change of the trailing leg is calculated by

4 $$\begin{aligned} \Delta \textbf{P}_\text {TL} = \left( \begin{array}{c} \Delta p_\text {x} \\ \Delta p_\text {y} \end{array}\right) = \textbf{P}_\text {TL}\,(t_\text {a}) - \textbf{P}_\text {TL}\,(t_\text {to}) \hspace{0.25cm} \text {with} \hspace{0.25cm} \textbf{P}_\text {TL}\,(t) = \sum _{i=1}^{3} m_i \textbf{v}\,(t) = \sum _{i=1}^{3} m_i \left( \begin{array}{c} v_{\text {x}, i}\,(t) \\ v_{\text {y}, i}\,(t) \end{array}\right) \,, \end{aligned}$$

where $m_i$ are the masses of the three segments of the trailing leg; the foot, shank, and thigh. $v_{\text {x},i}$ and $v_{\text {y},i}$ are the center of mass velocities at $t_\text {a}$ and $t_\text {to}$ of the respective segments (x- and y-directions). We only consider samples with $t_\text {a}$ after 35% stride as zero crossings before 35% are non-physiological. We also computed the angle $\Delta \alpha _p$ between the momentum vectors at the beginning of the alleviation phase $\textbf{P}_\text {TL}\,(t_\text {a})$ and TO $\textbf{P}_\text {TL}\,(t_\text {to})$ with

5 $$\begin{aligned} \Delta \alpha _p = \cos ^{-1} \left( \frac{\textbf{P}_\text {TL}\,(t_\text {a}) \cdot \textbf{P}_\text {TL}\,(t_\text {to})}{\left| \textbf{P}_\text {TL}\,(t_\text {a})\right| \cdot \left| \textbf{P}_\text {TL}\,(t_\text {to})\right| } \right) . \end{aligned}$$

For a visualization of the momentum vectors and the momentum change during push-off, see Ref.^7^, Fig. 4.

Finally, we evaluated several global gait characteristics to assess the model’s overall walking behavior. The quantities included the average forward walking speed of the model $\bar{v}_{x}$, the average metabolic power consumption normalized to body weight $P_\text {metab}$ based on^33^, the stride time $t_\text {stride}$, the step length $l_\text {step}$, and the duty factor (DF), that is, how much of the stride time the foot is in contact with the ground. All values were measured from the last successful stride of the simulation, assuming stationary, symmetric walking.

To explore the influence of GRFs on the push-off process and to investigate the timing of gait events, we selected three trials from the systematic search for detailed analysis. For each search grid in which the GAS, VAS, or HAM were deactivated, we selected the trial with the maximum stable duration of muscle deactivation during preswing (50-62% of the gait cycle according to^13^). The selection is based on the assumption that the longest period of deactivation will induce the most pronounced changes, providing insight into the role of the muscles.

## Results

We investigated how rapid the knee flexion associated with the ankle push-off initiation is influenced by the muscles spanning the knee joint, including the biarticular muscles GAS and HAM. We tested when in the gait cycle and for how long GAS, HAM, and VAS could individually be deactivated while maintaining steady-state walking and impulsive ankle push-off. By assessing the changes in gait kinematics, kinetics, and GRFs in the absence of the muscles, we were able to identify the role of each muscle for knee flexion and ankle push-off.

### Deactivated GAS

**Fig. 2:**
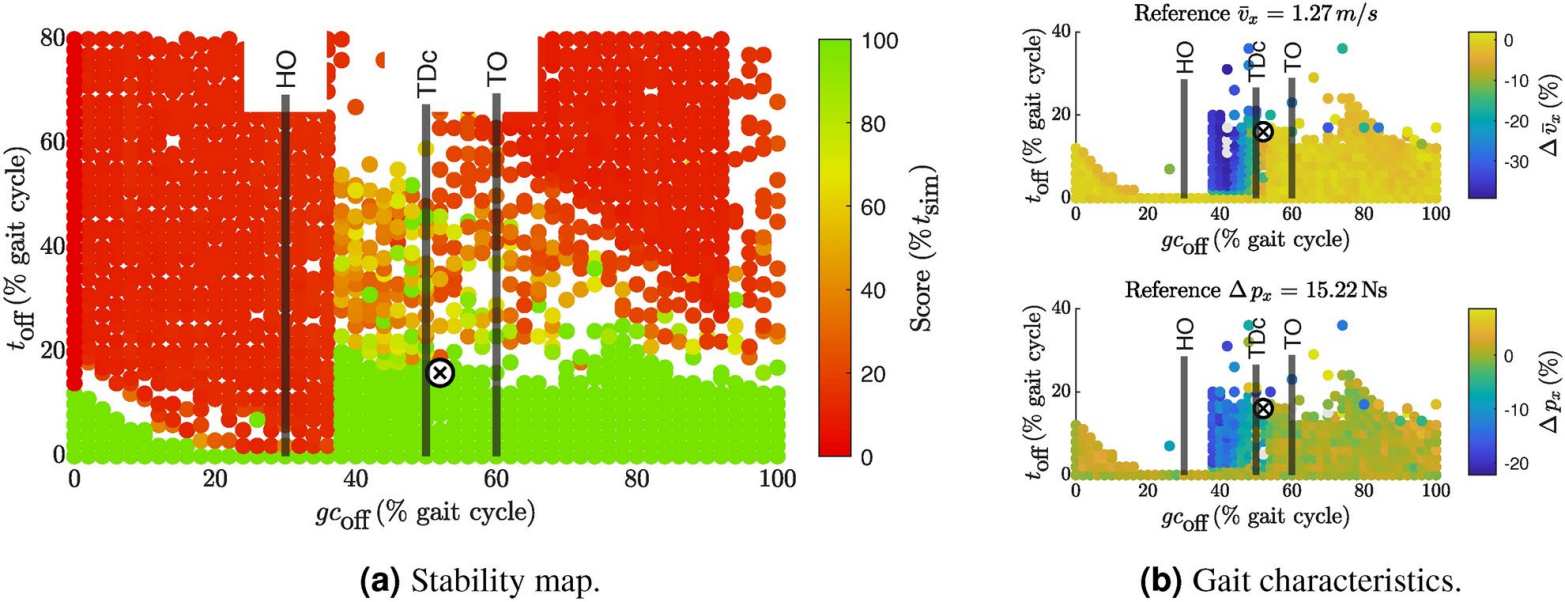
Scatter plot for stability score in (a) and selected gait characteristics when turning off GAS. In (**a**), only simulations that produced valid stride-normalized results are shown. Due to the normalization of $t_\text {off}$ to % stride time, most failed simulations fall outside the displayed y-axis limits. The failed samples often appear higher up on the y-axis due to post-processing errors in step detection when the model falls. White areas indicate regions without data points. (**b**) shows the average forward walking velocity (top) and the *x*-momentum change (bottom) of the trailing leg. In (**b**), the colors represent the relative change to the reference solution. The absolute value for the reference quantity is given in the title of each scatterplot. The color scale includes the 1st to 99th percentile of all results, with outliers shown in gray. Note that the y-axis limits in (**b**) are different from those in (**a**), to focus on the range of viable solutions. The cross $\textcircled {x}$ marks the selected sample, which will be evaluated in detail. The vertical lines indicate the average timing of HO, TDc, and TO in human gait from^13^ for orientation. See Fig. S2 for scatter plots of all other metrics.

 The results of the systematic search when deactivating the GAS are shown in the scatter plots in Fig. 2. $t_\text {off}$ is normalized to stride time. Since the stride times vary from trial to trial, the regular grid in which the search was performed disappears when normalizing $t_\text {off}$ to stride time.

Turning off GAS was possible starting from 38% of the gait cycle for up to 20% of the total stride time, see green area in Fig. 2a. Some trials even exhibited viable gaits with GAS turned off for more than 50% of the gait cycle. However, the green outliers far above the green area are likely to be error prone. If the model is close to falling, estimating the stride time may give misleading results because it is based on the evaluation of the last successful step. Figure 2b shows how the velocity and x-momentum of the trailing leg change when GAS is off. The largest changes occured when GAS was deactivated after 38% of the gait cycle and before TDc. The earlier GAS was turned off, the more pronounced the changes were with more than 30% reduction in walking speed (Fig. 2b). The forward momentum change of the trailing leg during push-off was reduced by up to 20%, indicating a reduced acceleration of the leg into swing. The trend towards a less dynamic gait was consistent with results of other evaluated metrics (see Fig. S2).

When GAS was turned off immediately after TDc, less changes of 10-20% decrease in walking speed and about 5% decrease in x-momentum change were visible (Fig. 2b). A deactivation after 50% of the gait cycle and in swing no longer yielded changes. From human data^13^ and the reference simulation^29^ it is known that GAS and SOL activity decreases strongly after TDc. Since GAS is inactive after push-off and throughout the swing phase, no changes were expected when deactivating GAS after TDc. For muscle activation and individual muscle contributions to joint torque in the reference solution, see FigS.. S1 and S5.

### **Deactivated VAS**

**Fig. 3:**
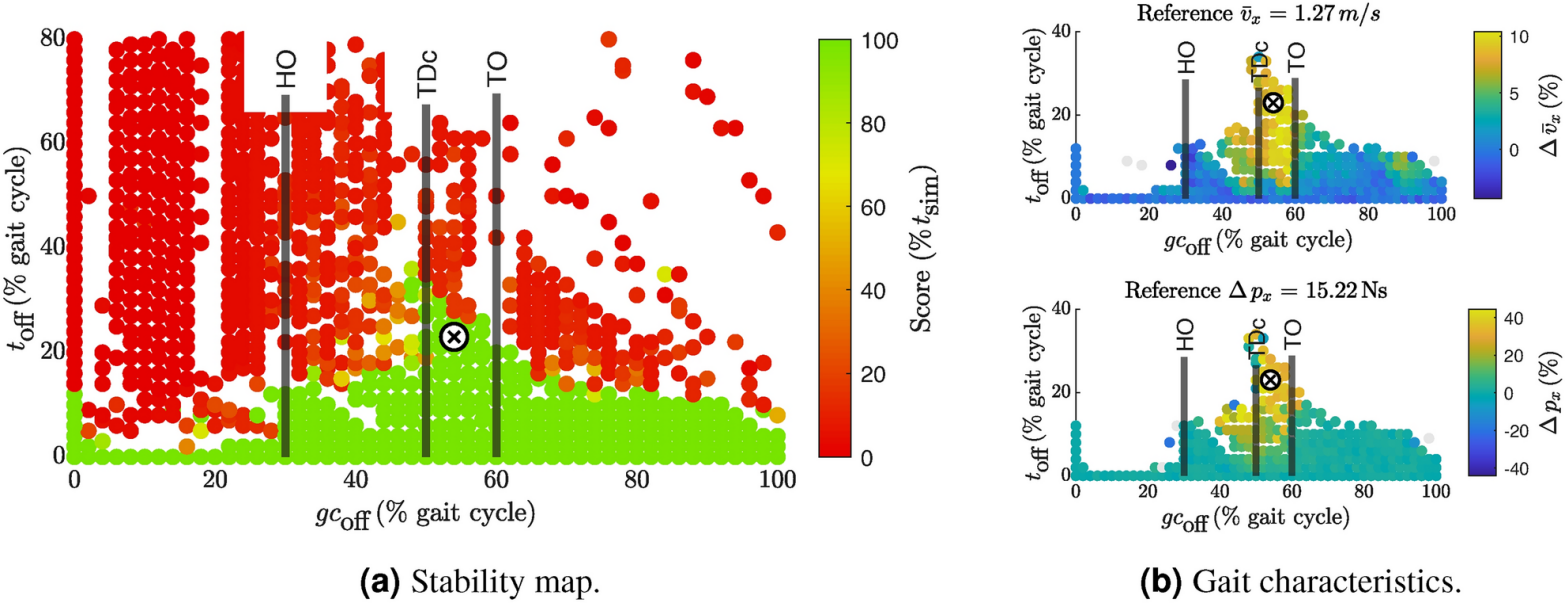
Scatter plot for stability score in (**a**) and selected gait characteristics when turning off VAS. In (**a**), only simulations that produced valid stride-normalized results are shown. Due to the normalization of $t_\text {off}$ to % stride time, most failed simulations fall outside the displayed y-axis limits. The failed samples often appear higher up on the y-axis due to post-processing errors in step detection when the model falls. White areas indicate regions without data points. (**b**) shows the average forward walking velocity (top) and the *x*-momentum change (bottom) of the trailing leg. In (**b**), the color represents the relative change to the reference solution. The absolute value for the reference quantity is given in the title of each scatterplot. The color scale includes the 1st to 99th percentile of all results, with outliers shown in gray. Note that the y-axis limits in (**b**) are different from those in (**a**), to focus on the range of viable solutions. The cross $\textcircled {x}$ marks the selected sample, which will be evaluated in detail. The vertical lines indicate the average timing of HO, TDc, and TO in human gait from Ref.^13^ for orientation. See Fig. S4 for scatter plots of all other metrics.

 Figure 3a shows the results of deactivating VAS. Viable solutions when deactivating VASexisted for up to 35% of the stride time between 45-60% of the gait cycle. The resulting gait pattern showed around 10% increase in walking speed (Fig. 3b top) and increased swing leg acceleration up to 45-50% in x-direction momentum change for the trailing leg (Fig. 3b bottom). Deactivating VAS slightly before and during push-off thus resulted in a more dynamic gait, which is a consistent finding with the remaining metrics, as shown in Fig.. S4.

### Deactivated HAM

**Fig. 4:**
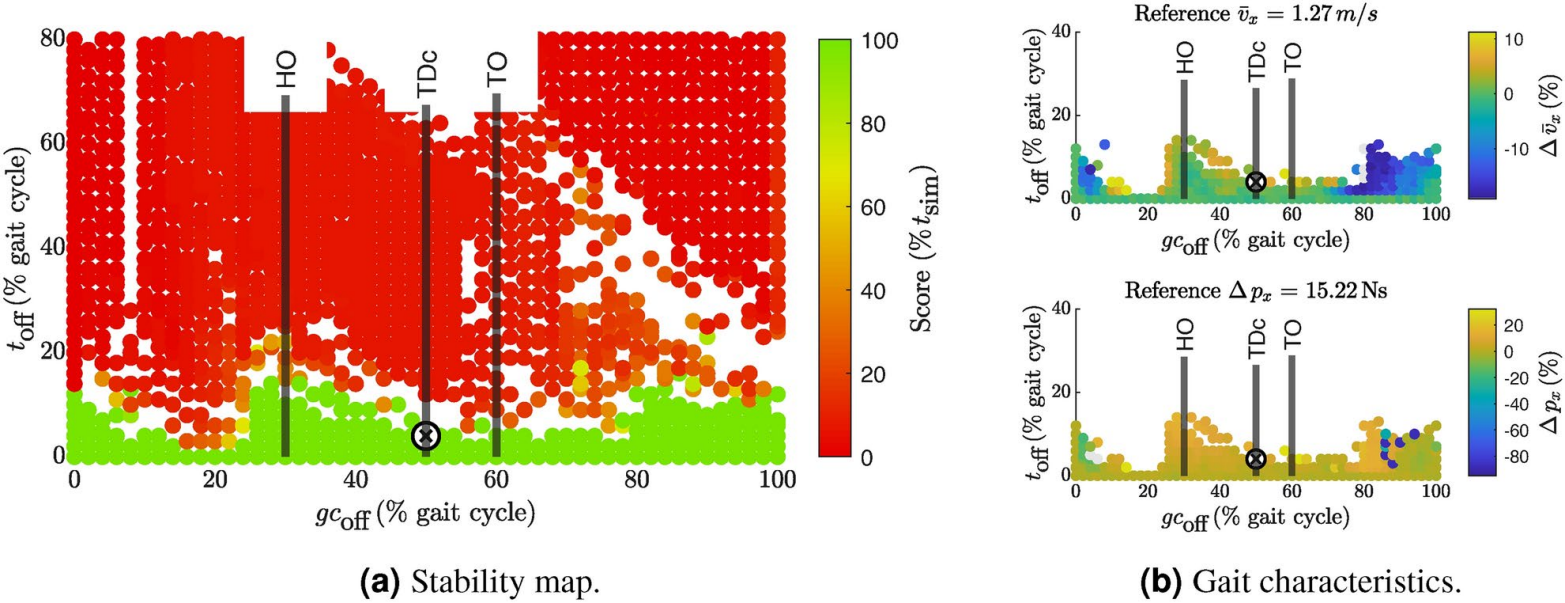
Scatter plot for stability score (**a**) and selected gait characteristics when turning off HAM (**b**). In (**a**), only simulations that produced valid stride-normalized results are shown. Due to the normalization of $t_\text {off}$ to % stride time, most failed simulations fall outside the displayed y-axis limits. The failed samples often appear higher up on the y-axis due to post-processing errors in step detection when the model falls. White areas indicate regions without data points. (**b**) shows the average forward walking velocity (top) and the *x*-momentum change (bottom) of the trailing leg. In (**b**), the color represents the relative change to the reference solution. The absolute value for the reference quantity is given in the title of each scatterplot. The color scale includes the 1st to 99th percentile of all results, with outliers shown in gray. Note that the y-axis limits in (**b**) are different from those in (**a**), to focus on the range of viable solutions. The cross $\textcircled {x}$ marks the selected sample, which will be evaluated in detail. The vertical lines indicate the average timing of HO, TDc, and TO in human gait from Ref.^13^ for orientation. During the relevant time window for ankle push-off, from 45% to 60% of the gait cycle, HAM deactivation shows almost no viable gaits. Changes in the evaluated gait characteristics mainly occur for deactivations at the end of swing. See Fig. S3 for scatter plots of all other metrics.

 Figure 4 shows the scatter plots with the results for the deactivation of HAM. Deactivation of more than 5% stride time was possible between 25-40% of the gait cycle and at the end of swing between 80-100% of the gait cycle. Changes in gait characteristics occurred mainly when HAM was deactivated during swing after 80%, see Fig. 4a. During the relevant time window for ankle push-off, from 45% to 60% of the gait cycle, HAM deactivation showed almost no viable gaits.

### Detailed analysis of selected simulations

 Table 1 summarizes the evaluated gait measures for the selected trials marked with crosses in Figs. 2,3 and 4. The table states the turn-off time and duration in parentheses after each trial name in the header. Since HAM could only be turned off for the short duration of 50 ms, the results were similar to the reference simulation. The deviation from the reference simulation was less than 8% for all quantities. Therefore, HAM deactivation trials are not further discussed here. Instead, we refer to the appendix with plots for joint kinetics and kinematics and muscle activation with deactivated HAM in Fig. S1.

The selected trials with deactivated GAS and VAS showed pronounced changes compared to the reference simulation. A deactivation of GAS in preswing resulted in an 18% decrease in walking speed, an 8% increase in metabolic energy expenditure, and a 22% decrease in ankle power amplification. On the other hand, deactivation of VAS increased walking speed by 9% and ankle power amplification by 104% compared to the reference simulation. Thus, slack knee flexion without VAS activation benefited push-off and overall gait dynamics, resulting in faster walking with impulsive swing leg acceleration. Deactivating GAS impaired ankle push-off and overall gait dynamics.

**Table 1:**
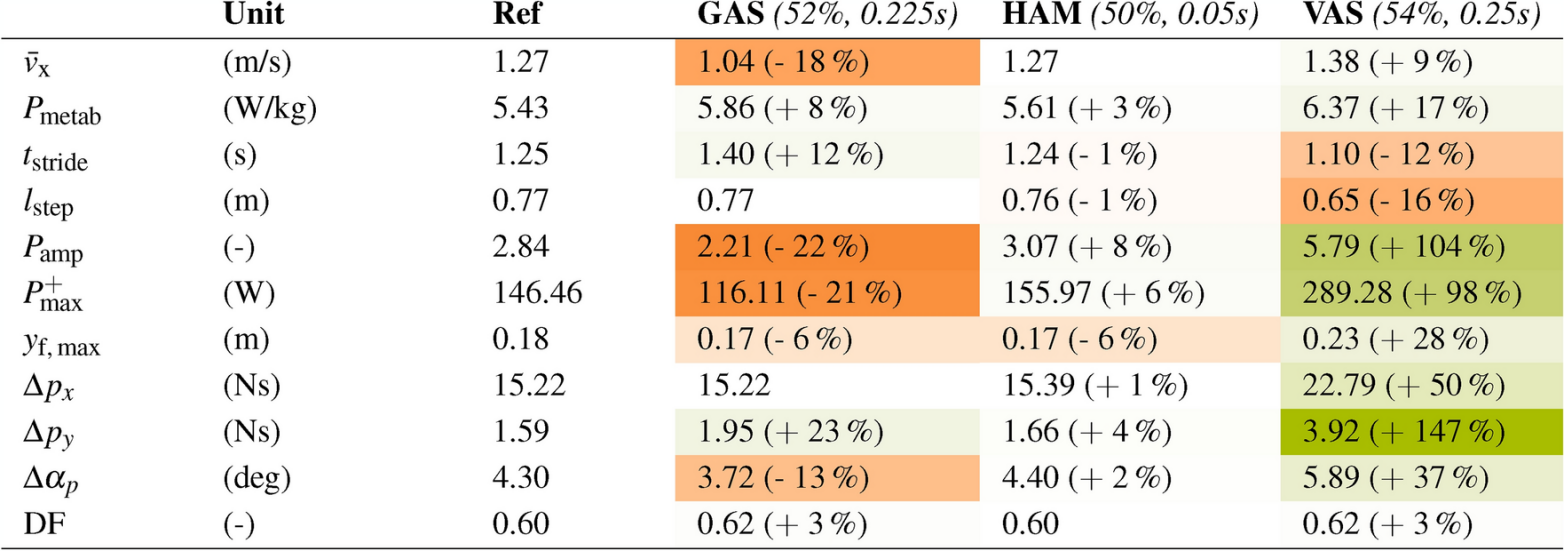
Summary of the evaluated gait measures for the selected trials.

The table summarizes the gait measures for the reference simulation and the three selected trials marked with a cross in figs. 4 to 3. The numbers in parentheses after the muscle names indicate when the muscle was deactivated in % of the gait cycle and how long it was inactive in seconds. The measures include: the forward walking speed $\bar{v}_{x}$; the metabolic power consumption $P_\text {metab}$; the stride time $t_\text {stride}$; the step length $l_\text {step}$; the ankle power amplification $P_\text {amp}$ according to^7^ defined in eq. (3); the maximum positive ankle peak power $P^+_\text {max}$; the maximum foot clearance in swing $y_\text {f,\,max}$; the momentum change $\Delta p_x$ and $\Delta p_y$ of the trailing leg from the beginning of alleviating phase to TO defined in eq. (4); the angle change of the momentum vector from the beginning of alleviation phase to TO $\Delta \alpha _p$; and the duty factor $DF = {t_\text {stance}}/{t_\text {stride}}$. The color intensity for each cell encodes its deviation from the reference simulation. Within each row $x_\text {row, ref}$, $I_\text {color} = {|(x_\text {cell} - x_\text {row, ref})|}/{x_\text {row, ref}} \in [0, 1]$. The colors show the direction of change, where green marks an increase compared to the reference and orange a decrease.

**Fig. 5:**
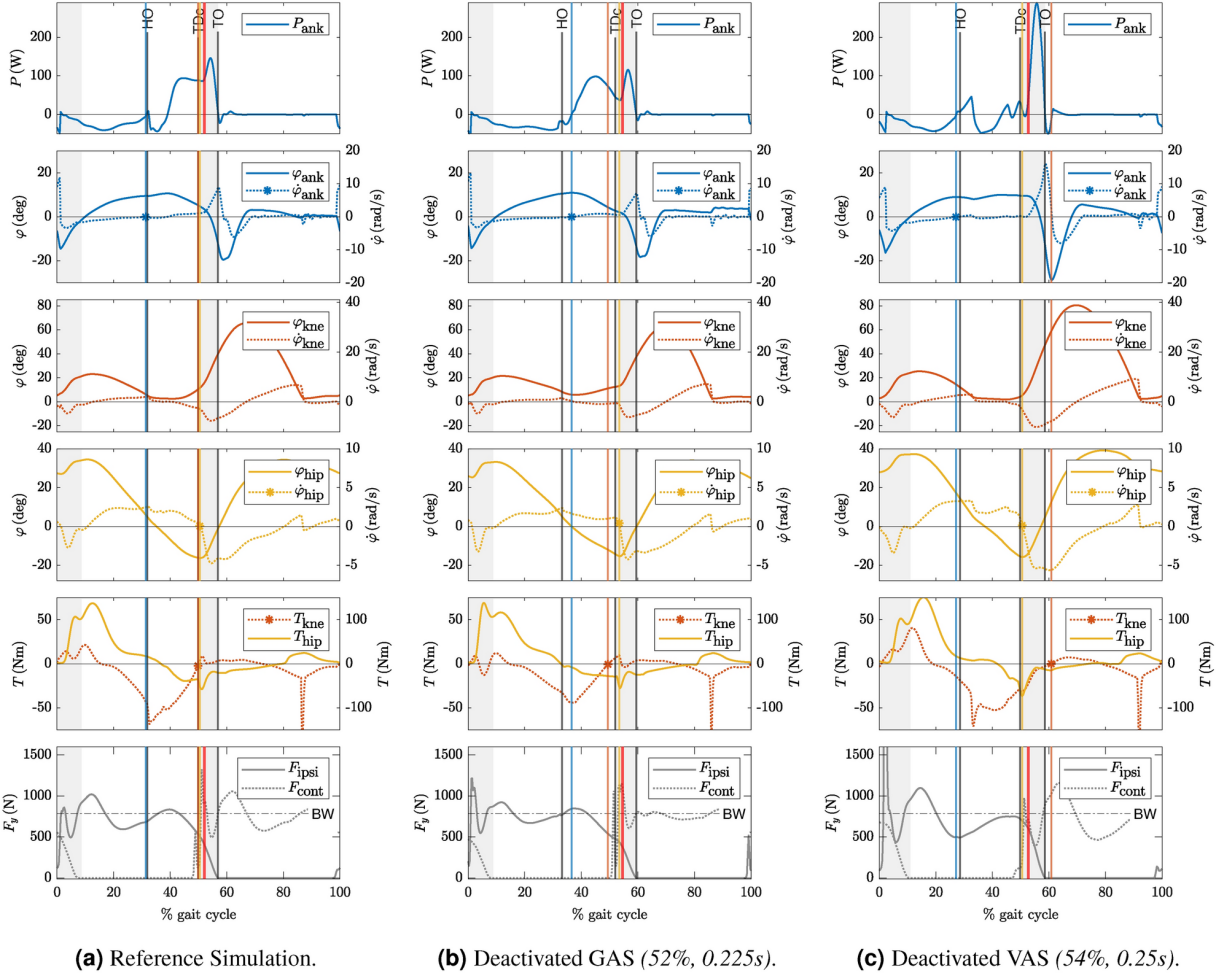
Timing of gait events around ankle push-off. Blue curves show ankle-related measurements, orange knee-related measurements, yellow hip-related measurements, and gray the vertical GRFs of the ipsi- and contralateral leg. The gray vertical lines show HO, TDc, and TO, respectively, as indicated in the ankle power plot at the top. The shaded areas indicate double support. HO marks the beginning of terminal stance. TDc marks the end of terminal stance and the beginning of preswing. TO marks the end of preswing and the beginning of the swing phase^13^. The blue vertical line marks the zero crossing (blue asterisk) of the ankle joint angular velocity, i.e., the point of maximum ankle dorsiflexion. The yellow vertical line marks the zero crossing (yellow asterisk) of the angular velocity of the hip joint in preswing, i.e., the start of hip flexion. The orange vertical line shows the zero crossing (orange asterisk) of the knee torque. The red vertical lines in the ankle power at the top and GRF plot at the bottom show the point of the last GRF snapshot given in Fig. 6 (e) and (j) for the reference simulation and deactivated VAS. The snapshots for GAS are provided in the appendix Fig. S7.

Figure 5 shows the timing of gait events. For each simulation, the figure shows the ankle power curve at the top, followed by the ankle, knee, and hip angles and angular velocities. The hip and knee joint torques and the vertical GRFs are shown at the bottom of each figure. The reference simulation is shown in (a), the simulation with deactivated GAS in (b), and the deactivated VAS in (c). The gray areas mark double support (DS), and the vertical lines show HO, TDc, and TO as transitions between gait phases^13^. In addition, we have included three events that are critical to push-off. The first event in the second line of plots is the onset of ankle plantarflexion, i.e., the zero crossing of the angular velocity of the ankle joints. The second event in the fourth line of plots is the onset of hip flexion, and the third event in the fifth line of plots is the zero crossing of the knee torque^7^.

In the reference simulation (Fig. 5a), the HO overlaped with the start of ankle plantarflexion. The start of hip flexion, the zero crossing of the knee moment, TDc, and the point of minimum hip torque, i.e., maximum flexion torque, coincided. When GAS and VAS were turned off, the start of ankle plantarflexion no longer coincided with HO. Instead, the plantarflexion started after HO for deactivated GAS, see Fig. 5b. With deactivated VAS, the ankle had an almost constant angle between HO and TDc. Because the angular velocity crosses zero several times, it was difficult to precisely determine when plantarflexion starts. In Fig. 5c, we chose to indicate the first zero crossing prior to HO. For the GAS trial in Fig. 5b, the knee torque became positive before TDc. For the deactivated VAS, the knee torque remained negative until the start of swing, see Fig. 5c. The zero-crossing of the knee torque occurred after TO when the power output at the ankle was already over.

The onset of hip flexion occurred at or immediately after TDc in all three setups (yellow vertical lines, Fig. 5). The beginning of hip flexion coincided with rapidly increasing knee flexion velocity. A few milliseconds after the start of hip flexion, the impulsive ankle power release began with the sharply rising power curve.

**Fig. 6:**
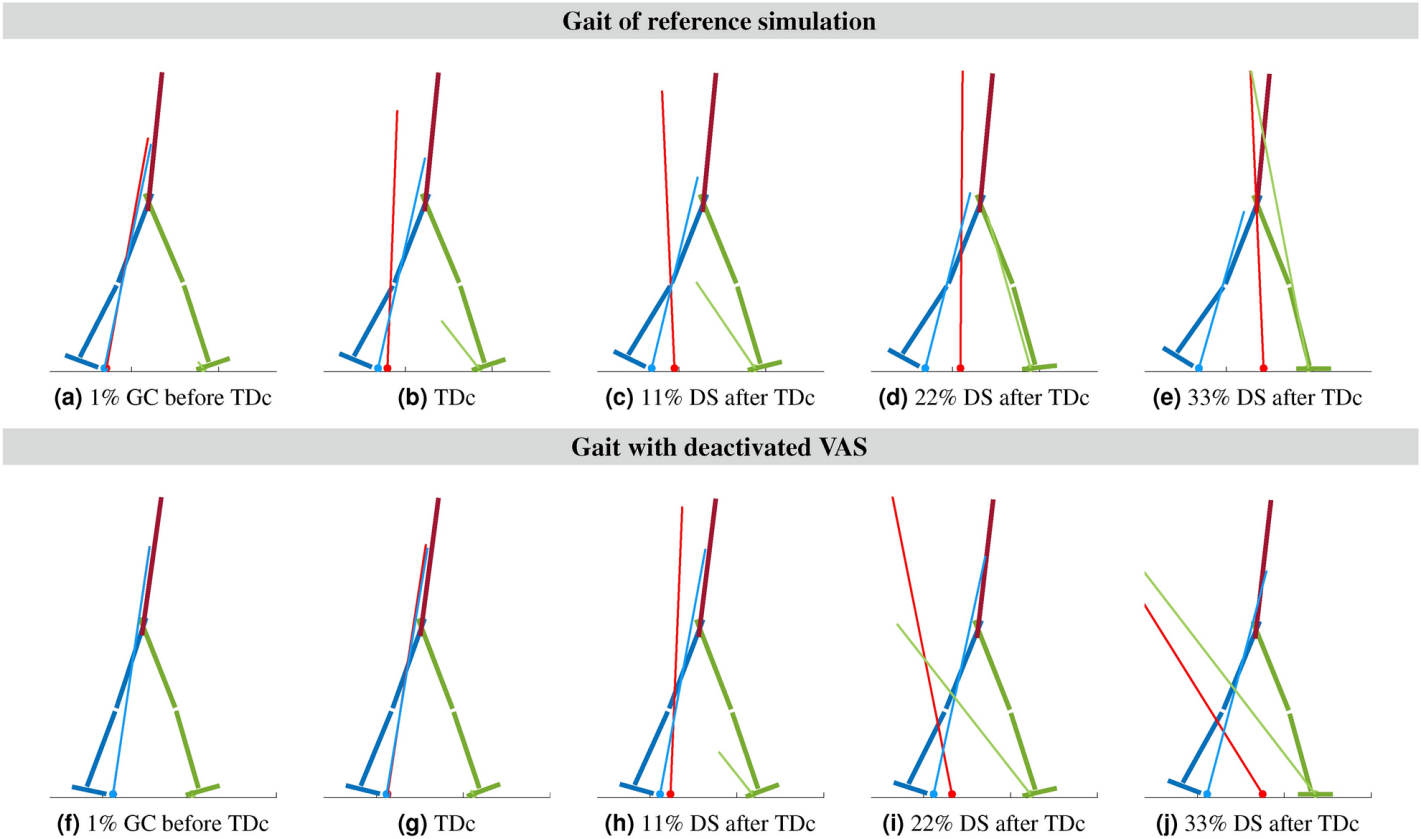
Visualization of the GRF vector around TDc. Red shows the global GRF vector, the blue and green vectors show the individual GRFs for each leg. For some snapshots with large GRFs due to the initial loading peak, the vectors’ length is truncated as only the orientation of the vectors is important for our analysis. Snapshots above in (**a**)-(**e**) show the reference simulation, below in (**f**)-(**j**) the trial with VAS turned off. The snapshots are taken at 1% of the gait cycle before TDc, at TDc, and in uniform steps at 11%, 22%, and 33% of the second DS phase after TDc. The GRF vectors originate from the global and foot-internal centers of pressure, respectively. For the reference simulation, the GRF vectors pass in front of the knee and behind the hip joint prior to TDc in (**a**). With TDc and afterward, in (**b**) and (**c**), the vectors pass through the neutral axis of the knee joint. As soon as the body weight is transferred to the contralateral leg in (**d**) and (**e**), the global GRF vector moves in front of the already buckled knee joint of the trailing leg. With the deactivated VAS, the GRF vectors pass in front of the neutral axis of the knee joint in (**f**) and (**h**). Snapshots for the GAS and HAM trials are available in Figs. S7 and S8.

Figure 6 shows snapshots of the model with the global and leg-specific GRF vectors before, during and after TDc for the reference simulation and the simulation with the deactivated VAS. The plots for deactivated HAM and GAS are provided in the appendix in Figs. S7 and S8. The time of the last snapshot was chosen by visually selecting the point just after the ankle power curve begins to rise sharply for the final power release. For all trials, as shown in the ankle power plot in Fig. 5, the power increase was observed at one-third of the duration of the second DS phase. The other snapshots are equally distributed between TDc and the final snapshot. The time of the last snapshot is marked by additional vertical red lines in the ankle power plots and the GRFs in Fig. 5.

Before TDc, the GRF vectors passed in front of the knee and behind the hip joint in both trials. The GRFs thus kept the leg straight by extending the knee. In the reference simulation, at TDc, and immediately after TDc, the vectors passed through the knee joint, possibly assisting knee flexion. When the weight was shifted to the contralateral leg in (e), as shown at bottom GRFs plot in Fig. 5a, the global GRF vector again passed in front of the knee. When VAS was deactivated, the GRF vectors behaved differently, see Fig. 6f-j. Unlike the reference simulation, the GRF vectors passed in front of the knee also after TDc. Only 22% of the DS phase after TDc, when the knee buckling had already started, the global GRF vector pointed behind the knee.

## Discussion

Our analysis aimed to determine whether active muscles are required for knee buckling in late stance at push-off initiation, and how the biarticular muscles GAS and HAM influence buckling. In addition, we investigated the extent to which GRFs drive knee buckling in terminal stance. We used a systematic parameter search to test when and for how long we could individually deactivate GAS, HAM, and VAS while maintaining steady-state walking. We then compared how the predicted gait, particularly the characteristics associated with impulsive ankle push-off and gait event timing around TDc, changed when the muscles were deactivated.

### Deactivating GAS

 We found viable gaits when deactivating VAS and GAS for up to 20% of a gait cycle starting slightly before or at TDc. A deactivated GAS before TDc between 38-50% of the gait cycle was found to be detrimental to gait efficiency, reducing power amplification and peak power during push-off (Fig. 5b). The model exhibited a less dynamic gait with reduced walking speed, shorter strides, reduced swing leg acceleration, and increased metabolic cost. Deactivation after 50%, i.e., the transition to pre-swing and push-off initiation, shows less change than when GAS is deactivated before TDc, but still emphasizes the benefits of having an active GAS at the start of push-off (Table 1, Fig. S2). The minor effect of GAS removal during late stance was expected, as EMG data show a rapid decline in muscle activity after 50% of the stride^13,34^. Our results are consistent with studies of pathological conditions like muscle weakness. Ong et al.^35^ showed that severe weakness in GAS and SOL resulted in slower gaits. An experimentally validated plantar flexor weakness simulation using the same reflex controller we used^29^ showed that 80% weakness in GAS and SOL reduced walking speed to 0.87 m/s, compared to 1.35 m/s in unimpaired individuals^36^. Disabling GAS in our study resulted in a slightly higher speed of 1.04m/s (see table 1), possibly due to compensatory effects of SOL. Energy cost analysis showed a 23% increase (from 4.21J/kg,m to 5.21J/kg,m) for weak plantar flexors in^36^, where our simulations showed an 8% increase (see Table 1). Experimental data also suggest reduced walking speed or compensatory hip and contralateral leg work for unilateral plantar flexor weakness^37^. Hemiparetic stroke studies further highlight paretic GAS and SOL to reduced forward propulsion and ankle power generation, impairing dynamic gait^38^. We conclude that an active GAS is required throughout the stance phase for coupling and synchronizing knee and ankle, but is not solely responsible for initiating knee buckling around TDc. The importance of GAS for ankle-knee joint synchronization has been demonstrated in several legged robots^15,20^. Although no active contraction is required, the GAS must, at minimum, withstand the forces resulting from the loading of the Achilles tendon^14^. We assume that the prolonged inactivity of the GAS in the stance phase impaired the ankle-knee coupling, which reduced walking performance.

### Deactivating VAS

 When VAS is deactivated, the TO happens before the zero-crossing of the knee torque, which is different from the reference simulation and the deactivated GAS. The swing leg is accelerated more into the vertical direction with 147% increase of $\Delta p_y$ and 28% increase of $y_\text {f,\,max}$ compared to the reference simulation (table 1). The ankle peak power is almost doubled.

In humans, the knee extensor VAS is mainly responsible for shock absorption and breaking during loading response and mid-stance between 0-30% of the gait cycle^13^. In the reference simulation, VAS is again activated between 45-55% of the gait cycle, see muscle activation in Fig. S1 and torque contributions in Fig. S5. The EMG patterns reported in literature^13,34^ show that VAS is usually inactive from 30-85% of the gait cycle. An exception is the VAS intermedius, which is sometimes activated at the end of stance 55-60% of the gait cycle^34^, which matches our results. Also, VAS activation between 45-55% occasionally happens in human individuals^39,40^, but is not generally reported^13^.

The importance of knee flexion without resistance from extensors during terminal stance and preswing is also known from pathologic stiff-knee gait associated with cerebral palsy and stroke^41,42^. A typical treatment for stiff-knee gait involves rectus femoris (RF) transfer, where the tendon is reattached to act as a flexor rather than an extensor, reducing the knee extension moments at push-off^43,44^. Forward dynamics simulations showed that iliopsoas and GAS activity during double support can increase knee flexion velocity, whereas VAS, RF, and SOL decrease it^42^. Consistent with our findings, a knee flexion without extensor resistance promotes a more dynamic and energy-efficient gait^42^. In contrast to our observations, women with knee osteoarthritis, exhibiting a 34% reduction in quadriceps torque, show longer support and step times, reduced swing times, and slower gait speeds compared to healthy individuals^45^. The difference to our experiments is that in osteoarthritis, the reduced VAS force persists throughout the entire gait cycle, not just around push-off. Thus, the aforementioned loading response is also impaired. In addition, pain affects gait dynamics^46^.

Allowing the knee to flex without VAS activation around push-off and early swing showed benefits of a dynamic gait with increased power amplification and peak ankle power compared to the reference simulation; see table 1 and Fig. 5c. Agostini et al.^40^ suggest that pre-swing VAS activation may modulate flexion velocity and increase patella stability for swing. A reduced flexion velocity when VAS is activated in pre-swing, as in the reference simulation, comes at the cost of a less dynamic gait, see table 1. The reduced gait dynamics may be why the activation is not reported in most healthy human subjects.

### Deactivating HAM

 Walking with deactivated HAM between 45-60% of the gait cycle was only feasible with short periods of deactivation below 50 ms. HAM deactivation severely reduced trunk pitch control, leading to unstable gaits. Van der Krogt et al.^47^ showed that gait was not robust to hip abductor and hip flexor weakness. However, weak hip and knee extensors could be better tolerated without causing large deviations from normal human gait. In contrast, our simulation was not robust to hip extensor weakness, which may be due to the fact that upper body stabilisation in our simulation differs significantly from human walking^48,49^. A complete absence of muscle strength is also different from weakness. It is, therefore, difficult to draw conclusions about the role of HAM in the push-off process from our experiments. In human walking, HAM activation is required around push-off to stabilize the upper body, as also described in^50^, to counteract the torques induced on the trunk by HFL contraction. Separating the effects of the upper body from the actual push-off process will require further investigation, e.g., by performing simulations with a fixed upper body which allows HAM to be deactivated for longer periods.

### Gait event timing and GRFs

 We found that the zero crossing of the knee torque, as discussed in^7^, does not necessarily indicate push-off initiation in our simulation. When VAS is deactivated, the zero crossing does not occur until the leg is in swing (Fig. 5c). However, this could also be an artifact of the simulation as the knee torque is generally more negative than human data; see torque data in Fig. S1.

In all our simulations, the GRF vector passes in front of the knee during single stance, keeping the knee straight without requiring an active muscle contribution to knee extension, as shown in Fig. 6. For the reference simulation, the GRF vector passes through the knee joint’s neutral axis with TDc and even behind the knee joint just after TDc. The knee configuration is now unstable, ready to buckle and launch the swing leg catapult. However, in the trials with VAS or GAS off, the GRF vector does not pass through or behind the knee until the impulsive ankle power output has already begun. So the knee flexion is likely forced by the hip torque, as both hip torque profiles match in shape and magnitude between 50-60% of the gait cycle. We, therefore, conclude that a purely geometric catapult release, as seen in horses ^25^, is unlikely for ankle push-off, at least in our simulation results.

The only gait event we found to coincide with push-off initiation across all trials was the onset of hip flexion. One possible mechanism to trigger impulsive ankle power release is active hip flexion around TDc. The hip flexion torque acting on the thigh of the trailing leg can cause the knee to buckle in response to the dynamic instability of the joint. The hip flexion torque thus “overdrives” the knee extension and forces the knee to buckle. The changed loading conditions at the end of the stance phase and the geometric arrangement of the GRFs near the knee joint’s neutral axis further support the rapid release of ankle power.

### Limitations

 The results discussed in this paper are based on a widely accepted and applied simulation model with reflex control architecture^29^. A simulation-based approach has limitations, and further research on human subjects, as in^7^, is needed to validate the results. Our model includes only seven muscles per leg and is restricted to 2D sagittal plane motion. It does not include the biarticular RF muscle that spans the knee and hip as an antagonist to HAM. RF also influences knee dynamics in stance and is often mentioned as necessary for postural stability along with HAM^50^.

The RF was not included in the model because we consider it to have limited relevance to the study’s focus on push-off dynamics. Although the RF is active during late stance (57-65% of the gait cycle^13^; 45-70% of the gait cycle^34^ ), its primary roles are described as knee extension control during swing, foot preparation for landing, and postural stability during the load response^13,51,52^. Significant contributions to push-off mechanics have not been reported to the authors’ knowledge. While the RF may affect details such as knee torque zero-crossings, we believe the main findings focused on passive knee buckling dynamics during push-off are unlikely to change.

In the reflex controller, the HFL is activated based on the landing of the contralateral leg^29^, II, C, p.265f and the forward tilt angle of the upper body^29^, II B p.265, while the HAM counteracts knee hyperextension in the case of large hip torques^29^, II B p.265. The suggestion that hip muscle activation is essential for the initiation of push-off thus depends on the given control architecture. Furthermore, controllers were not optimized for individual trials.

We chose not to reoptimize the controller to isolate the mechanical effects of muscle deactivation from the potentially confounding influence of neural adaptation, as demonstrated in^48^. In simulations of quadriceps weakness, Thompson et al.^53^ showed that compensatory activation of the gluteus maximus and SOL develops when healthy joint motion is enforced, and neural control adapts, which is the complementary method to our approach. Without reoptimizing the controller, the simulated response is likely to only partially represent the human response to muscle loss. We hypothesize that reoptimizing the controller would reduce the observed mechanical differences between the trials and the reference simulation. It is likely that the solution space for passive muscle configurations would expand, allowing muscles to be turned off for longer periods.

Besides sensory information from load receptors, i.e., the detection of the TDc that have been postulated for gait pattern generation in human walking^18^, other forms of sensory information would also be plausible, such as pre-flexes^54^, i.e., the anticipation of TDc, or hip flexor muscle stretch information^17^. The control concept of triggering the ankle push-off with the opposite leg’s touch down and with a slack knee flexion is attractive for prosthetic or robotic control design. Regardless of the exact function of the human push-off, our results can help reduce control effort and power consumption, and facilitate new design strategies for bipedal walking in machines and prostheses.

Our findings are currently limited to a generic neuromusculoskeletal model. However, there is significant variability between individuals in healthy human walking^55,56^.In particular, the net joint moments around the knee and the activation of the VAS muscle vary considerably^56^, which is particularly relevant to our observations of knee buckling and should be the subject of further investigation. In addition, gait and muscle recruitment patterns differ with walking speed^57–59^ and conditions such as inclined walking^60^, providing further opportunities for investigation to verify findings across conditions. Future work could also include the use of personalized models or control strategies^61^.

Setting the muscle stimulation to zero immediately at the defined turn-off time can cause the variable step-size integrator to adjust its step size. In some cases, changing the step size causes solver issues, such as convergence problems or instability, resulting in the simulation stopping, particularly in scenarios where the model is close to falling. Switching off muscle stimulation leads to a deactivation of the muscle according to the activation dynamics given in eq. (1). If the force decrease coincides for example with an impact event like touch-down (TD), the model may stumble, even though the model could have continued walking with the given passivity setting with a deactivation slightly before or after TD.

We estimate stride time based on the previous step. Hence, the switch-off timing may be inaccurate if the model stumbles. Variations in stride length or duration, or gait asymmetry within a simulation’s gait pattern due to muscle deactivation can lead to deviations between intended and actual deactivation points; see Fig. S1 for details. We evaluated our results based on the last successful step of each simulation. The model is assumed to exhibit periodic, stable walking until then. Therefore, we explicitly evaluated only the models with stable walking for the entire simulation time of 20 s.

Finally, the foot-ground contact model in the simulation affects the presented GRF analysis. A nonlinear spring-damper model inspired by^62,63^ is implemented in our neuromuscular simulation^29^ with a rigid foot and two contact points at the heel and ball of the foot. The GRFs show the characteristic double hump profile of human walking^29^. However, foot geometry and the mathematical contact formulation affect the GRF prediction^64,65^ and in combination with the optimization also the outcome of the gait predictions^66^. Further research is needed to validate the GRF analysis in human experiments and possibly with foot models of various complexity in simulation^49^.

## Conclusion

The importance of ankle push-off mechanics for human-like and efficient walking is widely recognized. Many prostheses and robotic devices already mimic push-off, either through active motors^67,68^ or elastic elements such as springs^5,15,69,70^. The purpose of this study was to determine whether active knee flexion through muscle activation is required for ankle push-off release. In addition, the role of the GRFs and the biarticular knee muscles GAS and HAM in knee buckling and push-off were investigated. We showed that entire muscle groups GAS and VAS can be deactivated for major parts of the stance phase and still produce viable gaits in simulation. The results provide insight into the observed variability in human gait and could be used to address a variety of other research questions, such as investigating compensatory mechanisms to for missing control or mechanics.

Our results show that deactivating VAS yields a more impulsive ankle push-off, resulting in a more dynamic gait pattern. Deactivating GAS, in contrast, yields reduced ankle peak power. Fast knee flexion followed by the impulsive ankle push-off, thus, favors an active GAS and a deactivated VAS to allow knee flexion without resistance from knee extension muscles. In all trials, the GRF vector aligns close to the knee joint’s neutral axis during push-off and assists knee flexion. However, the GRFs are unlikely to be independent drivers of knee flexion. Hip flexion torque in the pre-swing seems an additional factor in the push-off release, though this aspect will have to be proven in future work. Future research is required to validate the hypotheses in humans and through hardware testing on prostheses, exoskeletons, and robots. For engineering systems, our results highlight the importance of backdrivable actuators to allow fast knee flexion at swing initiation and the possibility of minimalist control of push-off timing based on the TDc.

## Supplementary Information

Supplementary Information.

## Data Availability

All data and code related to this manuscript are available in the following GitHub repository: https://gitlab.lrz.de/ecowalk/knee-buckling-and-ankle-push-off Human data from van der Zee et al.^71^ that we used for comparison are available at: https://github.com/timvanderzee/human-walking-biomechanics

## References

[1] Gulati, S. & Sondhi, V. Cerebral palsy: An overview. *Indian J. Pediatr.* **85**, 1006–1016. 10.1007/s12098-017-2475-1 (2018). 29152685 10.1007/s12098-017-2475-1

[2] Song, S. & Geyer, H. Predictive neuromechanical simulations indicate why walking performance declines with ageing. *J. Physiol.* **596**, 1199–1210. 10.1113/JP275166 (2018). 29344967 10.1113/JP275166 PMC5878225

[3] Conway, K. A. & Franz, J. R. Shorter gastrocnemius fascicle lengths in older adults associate with worse capacity to enhance push-off intensity in walking. *Gait Posture* **77**, 89–94. 10.1016/j.gaitpost.2020.01.018 (2020). 32004951 10.1016/j.gaitpost.2020.01.018 PMC7479307

[4] Renjewski, D., Lipfert, S. W. & Günther, M. Foot function enabled by human walking dynamics. *Phys. Rev. E* **106**, 064405. 10.1103/PhysRevE.106.064405 (2022). 36671109 10.1103/PhysRevE.106.064405

[5] Sawicki, G. S., Lewis, C. L. & Ferris, D. P. It pays to have a spring in your step. *Exerc. Sport Sci. Rev.* **37**, 130–138. 10.1097/JES.0b013e31819c2df6 (2009). 19550204 10.1097/JES.0b013e31819c2df6 PMC2821187

[6] Zelik, K. E., Huang, T.-W.P., Adamczyk, P. G. & Kuo, A. D. The role of series ankle elasticity in bipedal walking. *J. Theor. Biol.* **346**, 75–85. 10.1016/j.jtbi.2013.12.014 (2014). 24365635 10.1016/j.jtbi.2013.12.014 PMC3947753

[7] Lipfert, S. W., Günther, M., Renjewski, D. & Seyfarth, A. Impulsive ankle push-off powers leg swing in human walking. *J. Exp. Biol.* **217**, 1218–1228. 10.1242/jeb.097345 (2014). 24363410 10.1242/jeb.097345

[8] Hof, A. L., Geelen, B. A. & van den Berg, J. Calf muscle moment, work and efficiency in level walking; role of series elasticity. *J. Biomech.* **16**, 523–537. 10.1016/0021-9290(83)90067-2 (1983). 6619170 10.1016/0021-9290(83)90067-2

[9] Alexander, M. R. & Bennet-Clark, H. C. Storage of elastic strain energy in muscle and other tissues. *Nature* **265**, 114–117. 10.1038/265114a0 (1977). 834252 10.1038/265114a0

[10] Fukunaga, T. et al. In vivo behaviour of human muscle tendon during walking. *Proc. Biol. Sci.* **268**, 229–233. 10.1098/rspb.2000.1361 (2001). 11217891 10.1098/rspb.2000.1361 PMC1088596

[11] Ishikawa, M., Komi, P. V., Grey, M. J., Lepola, V. & Bruggemann, G.-P. Muscle-tendon interaction and elastic energy usage in human walking. *J. Appl. Physiol.* **99**, 603–608. 10.1152/japplphysiol.00189.2005 (2005). 15845776 10.1152/japplphysiol.00189.2005

[12] Lichtwark, G. A., Bougoulias, K. & Wilson, A. M. Muscle fascicle and series elastic element length changes along the length of the human gastrocnemius during walking and running. *J. Biomech.* **40**, 157–164. 10.1016/j.jbiomech.2005.10.035 (2007). 16364330 10.1016/j.jbiomech.2005.10.035

[13] Perry, J., Burnfield, J. M. & Cabico, L. M. *Gait Analysis: Normal and Pathological Function* (SLACK, 2010).

[14] Buchmann, A., Kiss, B., Badri-Spröwitz, A. T. & Renjewski, D. Power to the springs: Passive elements are sufficient to drive push-off in human walking. In *Robotics in Natural Settings*, 21–32, 10.1007/978-3-031-15226-9_5 (Springer International Publishing, 2023).

[15] Kiss, B. *et al.* Gastrocnemius and power amplifier soleus spring-tendons achieve fast human-like walking in a bipedal robot. In *2022 IEEE/RSJ International Conference on Intelligent Robots and Systems (IROS)*, 5202–5209, 10.1109/IROS47612.2022.9981725 (IEEE, 2022).

[16] Malcolm, P., Quesada, R. E., Caputo, J. M. & Collins, S. H. The influence of push-off timing in a robotic ankle-foot prosthesis on the energetics and mechanics of walking. *J. Neuroeng. Rehabil.* **12**, 21. 10.1186/s12984-015-0014-8 (2015). 25889201 10.1186/s12984-015-0014-8 PMC4404655

[17] Dietz, V. & Harkema, S. J. Locomotor activity in spinal cord-injured persons. *J. Appl. Physiol.* **96**, 1954–1960. 10.1152/japplphysiol.00942.2003 (2004). 15075315 10.1152/japplphysiol.00942.2003

[18] Pang, M. Y. C. & Yang, J. F. Sensory gating for the initiation of the swing phase in different directions of human infant stepping. *J. Neurosci.* **22**, 5734–5740. 10.1523/JNEUROSCI.22-13-05734.2002 (2002). 12097526 10.1523/JNEUROSCI.22-13-05734.2002 PMC6758226

[19] Seyfarth, A., Günther, M. & Blickhan, R. Stable operation of an elastic three-segment leg. *Biol. Cybern.* **84**, 365–382. 10.1007/PL00007982 (2001). 11357549 10.1007/PL00007982

[20] Schumacher, C., Sharbafi, M. A., Seyfarth, A. & Rode, C. Biarticular muscles in light of template models, experiments and robotics: a review. *J. R. Soc. Interface* **17**, 20180413. 10.1098/rsif.2018.0413 (2020). 32093540 10.1098/rsif.2018.0413 PMC7061696

[21] Malcolm, P., Galle, S., Derave, W. & de Clercq, D. Bi-articular knee-ankle-foot exoskeleton produces higher metabolic cost reduction than weight-matched mono-articular exoskeleton. *Front. Neurosci.* **12**, 69. 10.3389/fnins.2018.00069 (2018). 29551959 10.3389/fnins.2018.00069 PMC5841020

[22] Bianco, N. A., Franks, P. W., Hicks, J. L. & Delp, S. L. Coupled exoskeleton assistance simplifies control and maintains metabolic benefits: A simulation study. *PLoS One* **17**, e0261318. 10.1371/journal.pone.0261318 (2022). 34986191 10.1371/journal.pone.0261318 PMC8730392

[23] Unal, R. *et al.* Towards a fully passive transfemoral prosthesis for normal walking. In *2012 4th IEEE RAS & EMBS International Conference on Biomedical Robotics and Biomechatronics (BioRob 2012)*, 1949–1954, 10.1109/BioRob.2012.6290837 (IEEE, 2012).

[24] Gronenberg, W. Fast actions in small animals: Springs and click mechanisms. *J. Comp. Physiol. A*[SPACE]10.1007/bf00225821 (1996).

[25] Wilson, A. M., Watson, J. C. & Lichtwark, G. A. A catapult action for rapid limb protraction. *Nature* **421**, 35–36. 10.1038/421035a (2003). 12511944 10.1038/421035a

[26] Gronenberg, W. The fast mandible strike in the trap-jaw ant odontomachus. *J. Comp. Physiol. A* **176**, 399–408. 10.1007/BF00219065 (1995).

[27] Bennet-Clark, H. C. & Lucey, E. C. A. The jump of the flea: A study of the energetics and a model of the mechanism. *J. Exp. Biol.* **47**, 59–76. 10.1242/jeb.47.1.59 (1967). 6058981 10.1242/jeb.47.1.59

[28] Aerts, P., Osse, J. W. M. & Verraes, W. Model of jaw depression during feeding in astatotilapia elegans (teleostei: Cichlidae): Mechanisms for energy storage and triggering. *J. Morphol.* **194**, 85–109. 10.1002/jmor.1051940108 (1987). 29914223 10.1002/jmor.1051940108

[29] Geyer, H. & Herr, H. M. A muscle-reflex model that encodes principles of legged mechanics produces human walking dynamics and muscle activities. *IEEE Trans. Neural Syst. Rehabil. Eng.* **18**, 263–273. 10.1109/TNSRE.2010.2047592 (2010). 20378480 10.1109/TNSRE.2010.2047592

[30] Geyer, H., Seyfarth, A. & Blickhan, R. Positive force feedback in bouncing gaits?. *Proc. Biol. Sci.* **270**, 2173–2183. 10.1098/rspb.2003.2454 (2003). 14561282 10.1098/rspb.2003.2454 PMC1691493

[31] Hosea, M. E. & Shampine, L. F. Analysis and implementation of tr-bdf2. *Appl. Numer. Math.* **20**, 21–37. 10.1016/0168-9274(95)00115-8 (1996).

[32] Bank, R. E. et al. Transient simulation of silicon devices and circuits. *IEEE Trans. Comput. Aided Des. Integr. Circuits Syst.* **4**, 436–451. 10.1109/TCAD.1985.1270142 (1985).

[33] Umberger, B. R., Gerritsen, K. G. M. & Martin, P. E. A model of human muscle energy expenditure. *Comput. Methods Biomech. Biomed. Engin.* **6**, 99–111. 10.1080/1025584031000091678 (2003). 12745424 10.1080/1025584031000091678

[34] Tao, W., Liu, T., Zheng, R. & Feng, H. Gait analysis using wearable sensors. *Sensors* **12**, 2255–2283. 10.3390/s120202255 (2012). 22438763 10.3390/s120202255 PMC3304165

[35] Ong, C. F., Geijtenbeek, T., Hicks, J. L. & Delp, S. L. Predicting gait adaptations due to ankle plantarflexor muscle weakness and contracture using physics-based musculoskeletal simulations. *PLoS Comput. Biol.* **15**, e1006993. 10.1371/journal.pcbi.1006993 (2019). 31589597 10.1371/journal.pcbi.1006993 PMC6797212

[36] Waterval, N. F. J. et al. Validation of forward simulations to predict the effects of bilateral plantarflexor weakness on gait. *Gait Posture* **87**, 33–42. 10.1016/j.gaitpost.2021.04.020 (2021). 33882437 10.1016/j.gaitpost.2021.04.020

[37] Waterval, N. F. J., Brehm, M.-A., Ploeger, H. E., Nollet, F. & Harlaar, J. Compensations in lower limb joint work during walking in response to unilateral calf muscle weakness. *Gait Posture* **66**, 38–44. 10.1016/j.gaitpost.2018.08.016 (2018). 30145473 10.1016/j.gaitpost.2018.08.016

[38] Peterson, C. L., Hall, A. L., Kautz, S. A. & Neptune, R. R. Pre-swing deficits in forward propulsion, swing initiation and power generation by individual muscles during hemiparetic walking. *J. Biomech.* **43**, 2348–2355. 10.1016/j.jbiomech.2010.04.027 (2010). 20466377 10.1016/j.jbiomech.2010.04.027 PMC2922425

[39] Di Nardo, F. et al. Assessment of the variability of vastii myoelectric activity in young healthy females during walking: a statistical gait analysis. *J. Electromyogr. Kinesiol.* **25**, 800–807. 10.1016/j.jelekin.2015.07.004 (2015). 26198265 10.1016/j.jelekin.2015.07.004

[40] Agostini, V. et al. Normative emg activation patterns of school-age children during gait. *Gait Posture* **32**, 285–289. 10.1016/j.gaitpost.2010.06.024 (2010). 20692162 10.1016/j.gaitpost.2010.06.024

[41] Piazza, S. J. & Delp, S. L. The influence of muscles on knee flexion during the swing phase of gait. *J. Biomech.* **29**, 723–733. 10.1016/0021-9290(95)00144-1 (1996). 9147969 10.1016/0021-9290(95)00144-1

[42] Goldberg, S. R., Anderson, F. C., Pandy, M. G. & Delp, S. L. Muscles that influence knee flexion velocity in double support: implications for stiff-knee gait. *J. Biomech.* **37**, 1189–1196. 10.1016/j.jbiomech.2003.12.005 (2004). 15212924 10.1016/j.jbiomech.2003.12.005

[43] Gage, J. R., Perry, J., Hicks, R. R., Koop, S. & Werntz, J. R. Rectus femoris transfer to improve knee function of children with cerebral palsy. *Dev. Med. Child Neurol.* **29**, 159–166. 10.1111/j.1469-8749.1987.tb02131.x (1987). 3582786 10.1111/j.1469-8749.1987.tb02131.x

[44] Goldberg, S. R., Ounpuu, S., Arnold, A. S., Gage, J. R. & Delp, S. L. Kinematic and kinetic factors that correlate with improved knee flexion following treatment for stiff-knee gait. *J. Biomech.* **39**, 689–698. 10.1016/j.jbiomech.2005.01.015 (2006). 16439238 10.1016/j.jbiomech.2005.01.015

[45] Spinoso, D. H., Bellei, N. C., Marques, N. R. & Navega, M. T. Quadriceps muscle weakness influences the gait pattern in women with knee osteoarthritis. *Adv. Rheumatol.* **58**, 26. 10.1186/s42358-018-0027-7 (2018). 30657091 10.1186/s42358-018-0027-7

[46] Edmonds, D. W., McConnell, J., Ebert, J. R., Ackland, T. R. & Donnelly, C. J. Biomechanical, neuromuscular and knee pain effects following therapeutic knee taping among patients with knee osteoarthritis during walking gait. *Clin. Biomech.* **39**, 38–43. 10.1016/j.clinbiomech.2016.09.003 (2016). 10.1016/j.clinbiomech.2016.09.003 27654572

[47] van der Krogt, M. M., Delp, S. L. & Schwartz, M. H. How robust is human gait to muscle weakness?. *Gait Posture* **36**, 113–119. 10.1016/j.gaitpost.2012.01.017 (2012). 22386624 10.1016/j.gaitpost.2012.01.017 PMC4890623

[48] Buchmann, A. & Renjewski, D. An open-source framework for sensitivity analysis of predictive neuromuscular simulations: How muscle-tendon stiffness and tendon slack length affect push-off. In *2024 10th IEEE RAS/EMBS International Conference for Biomedical Robotics and Biomechatronics (BioRob)*, 94–101, 10.1109/BioRob60516.2024.10719766 (IEEE, 2024).

[49] Buchmann, A., Wenzler, S., Welte, L. & Renjewski, D. The effect of including a mobile arch, toe joint, and joint coupling on predictive neuromuscular simulations of human walking. *Sci. Rep.* **14**, 14879. 10.1038/s41598-024-65258-z (2024). 38937584 10.1038/s41598-024-65258-z PMC11211509

[50] Schumacher, C. et al. Biarticular muscles are most responsive to upper-body pitch perturbations in human standing. *Sci. Rep.* **9**, 14492. 10.1038/s41598-019-50995-3 (2019). 31601860 10.1038/s41598-019-50995-3 PMC6787002

[51] Annaswamy, T. M., Giddings, C. J., Della Croce, U. & Kerrigan, D. C. Rectus femoris: its role in normal gait. *Arch. Phys. Med. Rehabil.* **80**, 930–934. 10.1016/S0003-9993(99)90085-0 (1999). 10453770 10.1016/s0003-9993(99)90085-0

[52] Frigo, C. A., Wyss, C. & Brunner, R. The effects of the rectus femoris muscle on knee and foot kinematics during the swing phase of normal walking. *Appl. Sci.* **10**, 7881. 10.3390/app10217881 (2020).

[53] Thompson, J. A., Chaudhari, A. M. W., Schmitt, L. C., Best, T. M. & Siston, R. A. Gluteus maximus and soleus compensate for simulated quadriceps atrophy and activation failure during walking. *J. Biomech.* **46**, 2165–2172. 10.1016/j.jbiomech.2013.06.033 (2013). 23915576 10.1016/j.jbiomech.2013.06.033

[54] Araz, M. et al. Muscle preflex response to perturbations in locomotion: In vitro experiments and simulations with realistic boundary conditions. *Front. Bioeng. Biotechnol.* **11**, 1150170. 10.3389/fbioe.2023.1150170 (2023). 37214305 10.3389/fbioe.2023.1150170 PMC10194126

[55] Bianchi, L., Angelini, D. & Lacquaniti, F. Individual characteristics of human walking mechanics. *Pflug. Arch. Eur. J. Physiol.* **436**, 343–356. 10.1007/s004240050642 (1998). 10.1007/s004240050642 9644215

[56] Simonsen, E. B. & Alkjær, T. The variability problem of normal human walking. *Med. Eng. Phys.* **34**, 219–224. 10.1016/j.medengphy.2011.07.013 (2012). 21852174 10.1016/j.medengphy.2011.07.013

[57] Baroudi, L. et al. Investigating walking speed variability of young adults in the real world. *Gait Posture* **98**, 69–77. 10.1016/j.gaitpost.2022.08.012 (2022). 36057208 10.1016/j.gaitpost.2022.08.012

[58] Neptune, R. R., Sasaki, K. & Kautz, S. A. The effect of walking speed on muscle function and mechanical energetics. *Gait Posture* **28**, 135–143. 10.1016/j.gaitpost.2007.11.004 (2008). 18158246 10.1016/j.gaitpost.2007.11.004 PMC2409271

[59] den Otter, A., Geurts, A. C. H., Mulder, T. & Duysens, J. Speed related changes in muscle activity from normal to very slow walking speeds. *Gait Posture* **19**, 270–278. 10.1016/S0966-6362(03)00071-7 (2004). 15125916 10.1016/S0966-6362(03)00071-7

[60] Lichtwark, G. A. & Wilson, A. M. Interactions between the human gastrocnemius muscle and the achilles tendon during incline, level and decline locomotion. *J. Exp. Biol.* **209**, 4379–4388. 10.1242/jeb.02434 (2006). 17050853 10.1242/jeb.02434

[61] Kainz, H., Wesseling, M. & Jonkers, I. Generic scaled versus subject-specific models for the calculation of musculoskeletal loading in cerebral palsy gait: Effect of personalized musculoskeletal geometry outweighs the effect of personalized neural control. *Clin. Biomech.* **87**, 105402. 10.1016/j.clinbiomech.2021.105402 (2021). 10.1016/j.clinbiomech.2021.105402 34098149

[62] Scott, S. H. & Winter, D. A. Biomechanical model of the human foot: Kinematics and kinetics during the stance phase of walking. *J. Biomech.* **26**, 1091–1104. 10.1016/S0021-9290(05)80008-9 (1993). 8408091 10.1016/s0021-9290(05)80008-9

[63] Günther, M. & Ruder, H. Synthesis of two-dimensional human walking: A test of the lambda-model. *Biol. Cybern.* **89**, 89–106. 10.1007/s00422-003-0414-x (2003). 12905038 10.1007/s00422-003-0414-x

[64] Millard, M. & Mombaur, K. A quick turn of foot: Rigid foot-ground contact models for human motion prediction. *Front. Neurorobot.* **13**, 62. 10.3389/fnbot.2019.00062 (2019). 31440154 10.3389/fnbot.2019.00062 PMC6693511

[65] Shourijeh, M. S. & McPhee, J. Foot-ground contact modeling within human gait simulations: from kelvin-voigt to hyper-volumetric models. *Multibody Syst. Dyn.* **35**, 393–407. 10.1007/s11044-015-9467-6 (2015).

[66] Veerkamp, K. et al. Evaluating cost function criteria in predicting healthy gait. *J. Biomech.* **123**, 110530. 10.1016/j.jbiomech.2021.110530 (2021). 34034014 10.1016/j.jbiomech.2021.110530

[67] Grimmer, M. et al. A powered prosthetic ankle joint for walking and running. *Biomed. Eng. Online* **15**, 141. 10.1186/s12938-016-0286-7 (2016). 28105953 10.1186/s12938-016-0286-7 PMC5249039

[68] Bellman, R. D., Holgate, M. A. & Sugar, T. G. Sparky 3: Design of an active robotic ankle prosthesis with two actuated degrees of freedom using regenerative kinetics. In *2nd IEEE RAS & EMBS International Conference on Biomedical Robotics and Biomechatronics, 2008*, 511–516, 10.1109/BIOROB.2008.4762887 (IEEE, Piscataway, NJ, 2008).

[69] Brackx, B., van Damme, M., Matthys, A., Vanderborght, B. & Lefeber, D. Passive ankle-foot prosthesis prototype with extended push-off. *Int. J. Adv. Rob. Syst.* **10**, 101. 10.5772/55170 (2013).

[70] Naseri, A., Mohammadi Moghaddam, M., Grimmer, M. & Ahmad Sharbafi, M. Passive hydraulic prosthetic foot to improve the push-off during walking. *Mech. Mach. Theory* **172**, 104777. 10.1016/j.mechmachtheory.2022.104777 (2022).

[71] van der Zee, T. J., Mundinger, E. M. & Kuo, A. D. A biomechanics dataset of healthy human walking at various speeds, step lengths and step widths. *Sci. Data* **9**, 704. 10.1038/s41597-022-01817-1 (2022). 36385009 10.1038/s41597-022-01817-1 PMC9669008

