# Supplementary material for: How knee muscles and ground reaction forces shape knee buckling and ankle push-off in neuromuscular simulations of human walking
Source: Sci Rep. 2025 Jan 17;15:2249. doi: 10.1038/s41598-025-86147-z (PMC11742656; doi:10.1038/s41598-025-86147-z)
Supplement: Supplementary file 1 — Supplementary Information. [file 41598_2025_86147_MOESM1_ESM.pdf]

Supplementary Material to *"How Knee Muscles and Ground Reaction Forces Shape Knee Buckling and Ankle Push-off in Neuromuscular Simulations of Human Walking"*

Alexandra Buchmann[1,*], Bernadett Kiss[2], Alexander Badri-Spröwitz[2,3] and Daniel Renjewski[1]

[1] Chair of Applied Mechanics, Technical University of Munich, Germany
[2] Dynamic Locomotion Group, Max-Plank-Institute for Intelligent Systems, Stuttgart, Germany
[3] Department of Mechanical Engineering, KU Leuven, Belgium
[*] M.Sc., Corresponding author, E-Mail: alexandra.buchmann@tum.de

Contents

S1 Joint kinematics & kinetics, GRFs, ankle push-off, muscle activation & work loops . . . S2

S2 GAS: Heatmaps for walking speed, CoT, duty factor, stride time, step length, power amplification, ground clearance, and trailing leg momentum quantities . . . S3

S3 HAM: Heatmaps for walking speed, CoT, duty factor, stride time, step length, power amplification, ground clearance, and trailing leg momentum quantities . . . S4

S4 VAS: Heatmaps for walking speed, CoT, duty factor, stride time, step length, power amplification, ground clearance, and trailing leg momentum quantities . . . S5

S5 Single muscle contributions (SMC) to hip, knee and ankle torques for selected trials . . . S6

S6 Muscle specific mechanical energy contributions in stance for hip, knee, and ankle . . . S7

S7 Walking Model with GRF-Visualization . . . S8

S8 Gait Events and Ankle Push-off Release . . . S9

S1 Joint kinematics & kinetics, GRFs, ankle push-off, muscle activation & work loops

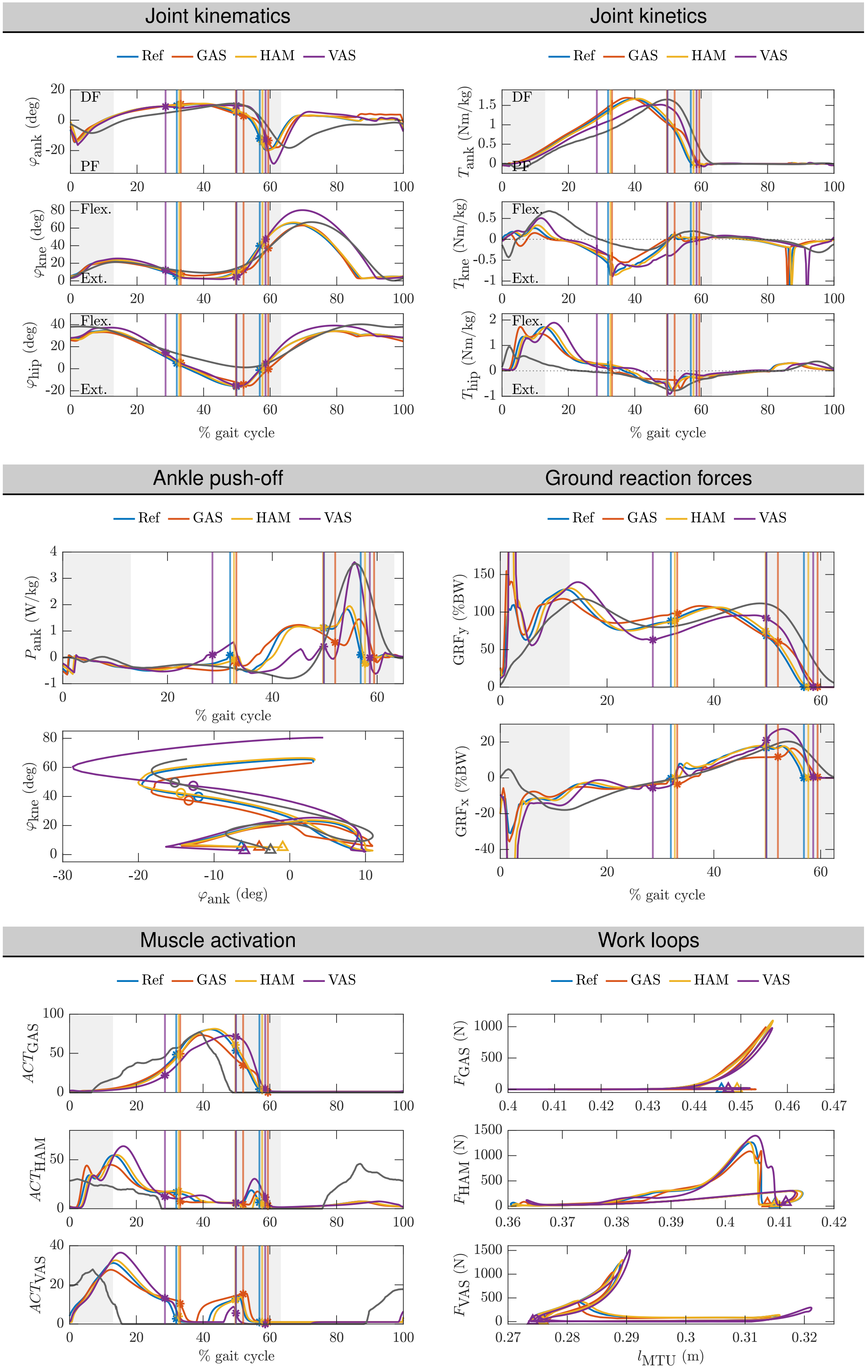

Figure S1: Joint kinematics, kinetics, ankle-knee coordination, ankle power, ground reaction forces (GRFs), muscle work loops and muscle activation for selected trials when the stimulation for selected muscles is turned off. Gray lines show human reference data from [1]. The selected trials for GAS, HAM and VAS are specified in table 1 and marked by crosses in figs. 2 to 4. Note that the intended turn-off point is not necessarily the actual one if the gait pattern is slightly irregular since muscle deactivation is based on the estimated stride time from the previous step.

S2 GAS: Heatmaps for walking speed, CoT, duty factor, stride time, step length, power amplification, ground clearance, and trailing leg momentum quantities

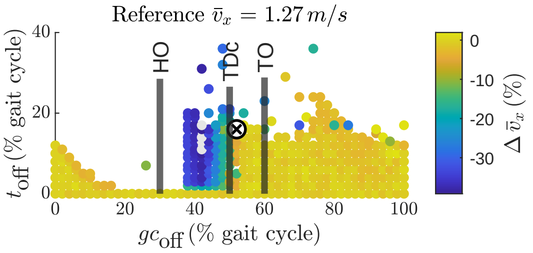

(a) Average forward walking velocity of head-arm-trunk (**HAT**).

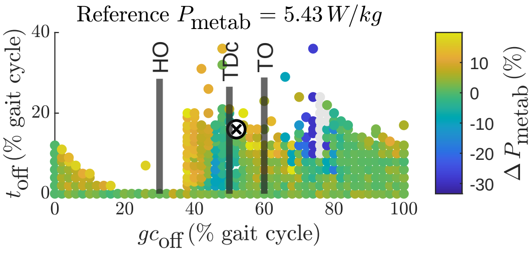

(b) Metabolic power consumption.

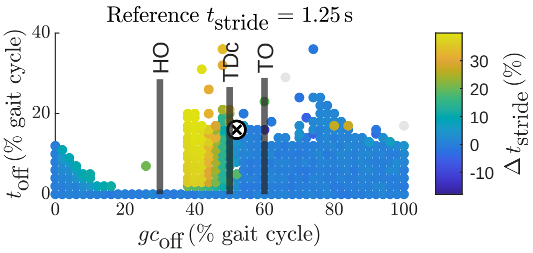

(c) Stride time.

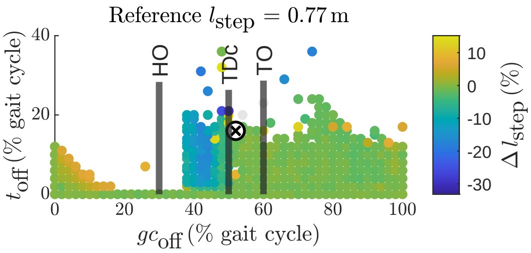

(d) Step length.

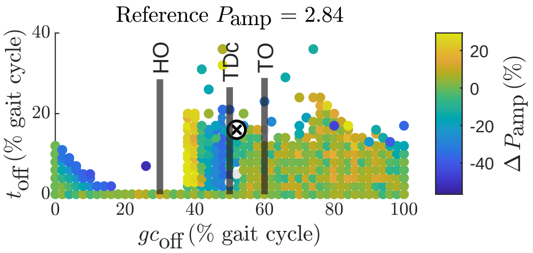

(e) Ankle power amplification.

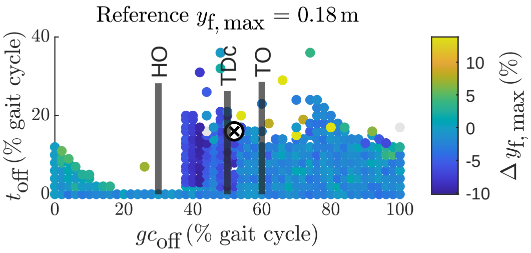

(f) Maximum foot-ground clearance.

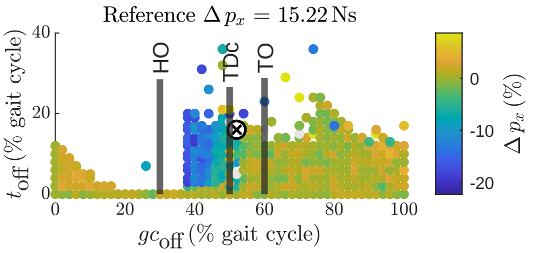

(g) Leg's momentum change in horizontal direction.

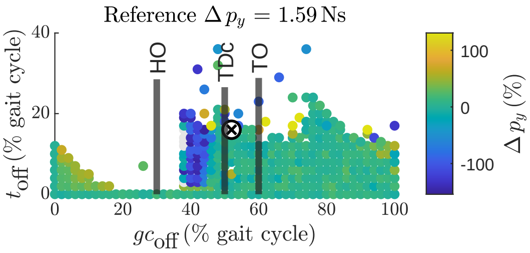

(h) Leg's momentum change in vertical direction.

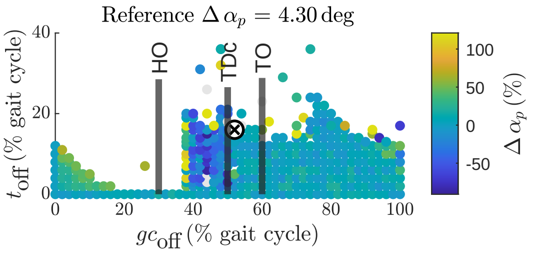

(i) Leg's momentum change angle from horizontal.

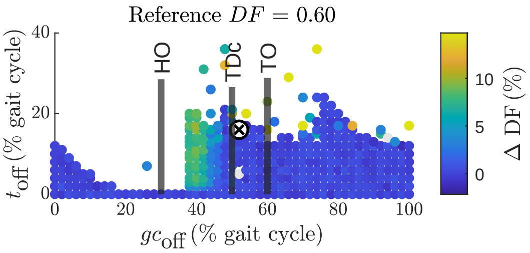

(j) Duty factor.

Figure S2: Heatmaps for turning off GAS stimulation. Outliers appear in gray and are defined as samples below 1% and above 99% percentiles of all results.

S3 HAM: Heatmaps for walking speed, CoT, duty factor, stride time, step length, power amplification, ground clearance, and trailing leg momentum quantities

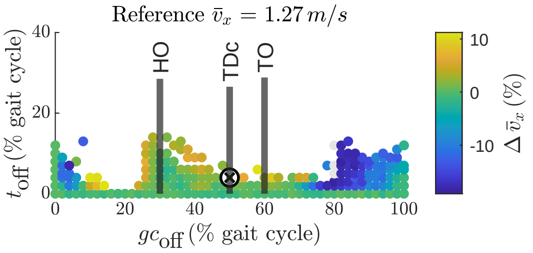

(a) Average forward walking velocity of head-arm-trunk (**HAT**).

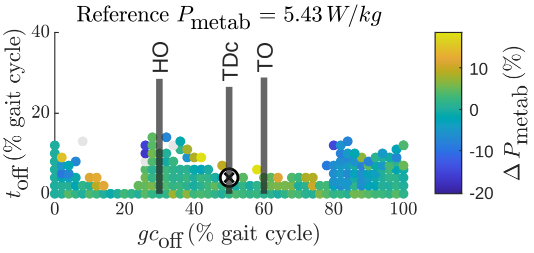

(b) Metabolic power consumption.

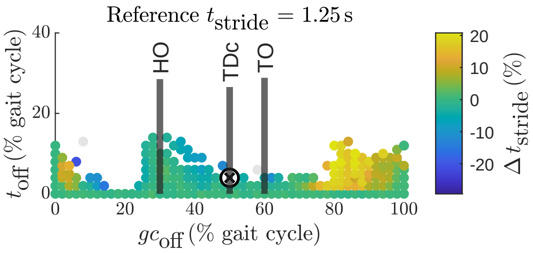

(c) Stride time.

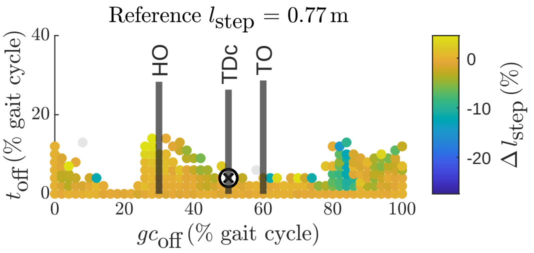

(d) Step length.

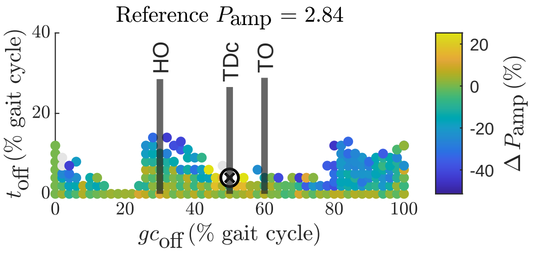

(e) Ankle power amplification.

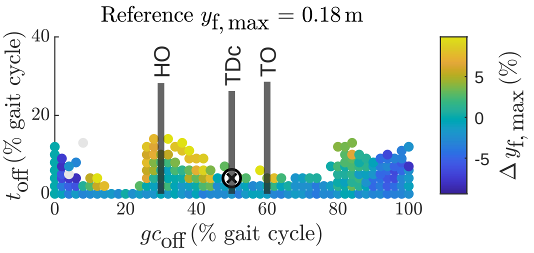

(f) Maximum foot-ground clearance.

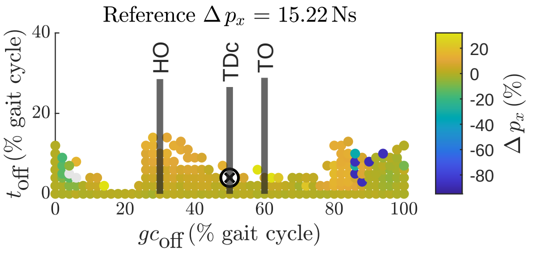

(g) Leg's momentum change in horizontal direction.

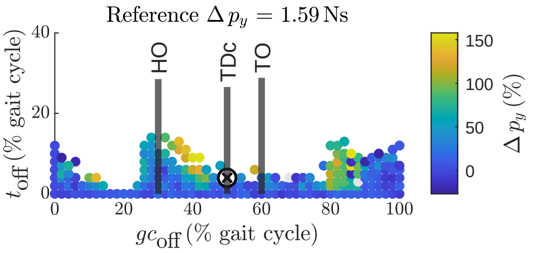

(h) Leg's momentum change in vertical direction.

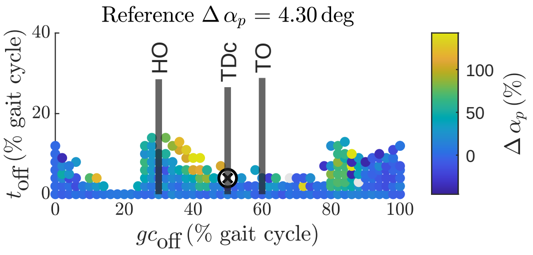

(i) Leg's momentum change angle from horizontal.

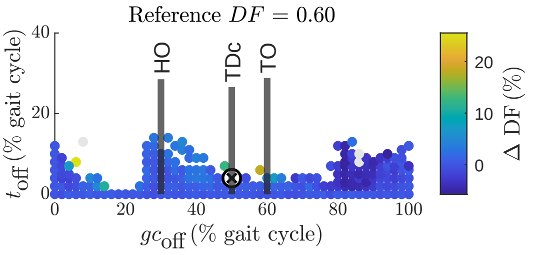

(j) Duty factor.

Figure S3: Heatmaps for turning off HAM stimulation. Outliers appear in gray and are defined as samples below 1% and above 99% percentiles of all results.

S4 VAS: Heatmaps for walking speed, CoT, duty factor, stride time, step length, power amplification, ground clearance, and trailing leg momentum quantities

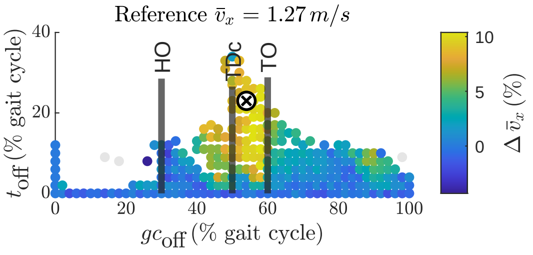

(a) Average forward walking velocity of head-arm-trunk (**HAT**).

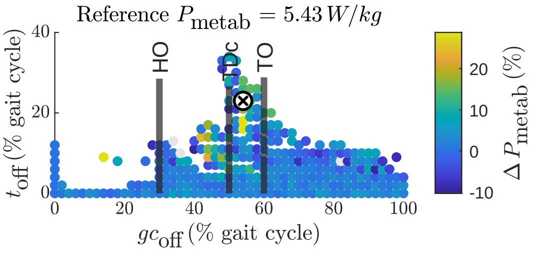

(b) Metabolic power consumption.

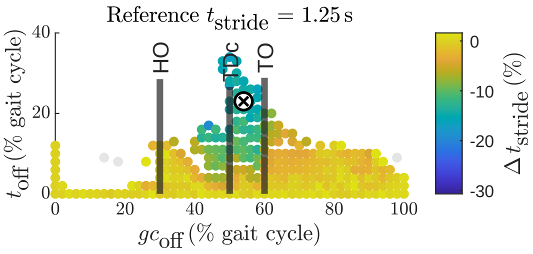

(c) Stride time.

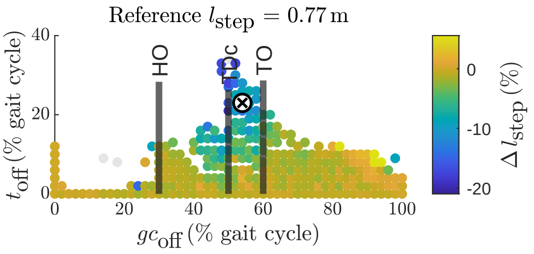

(d) Step length.

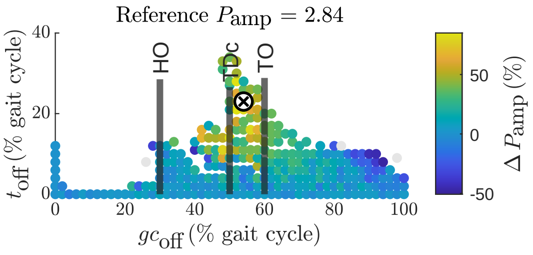

(e) Ankle power amplification.

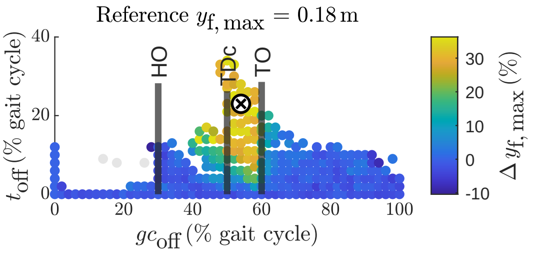

(f) Maximum foot-ground clearance.

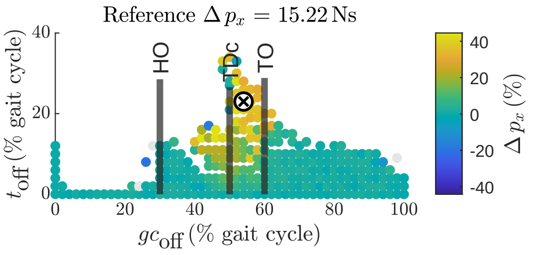

(g) Leg's momentum change in horizontal direction.

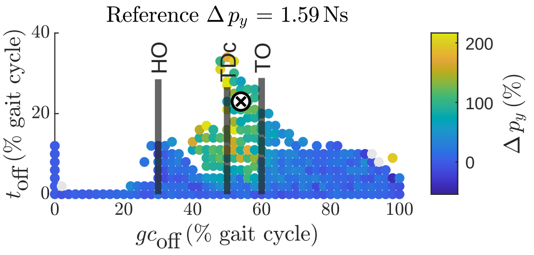

(h) Leg's momentum change in vertical direction.

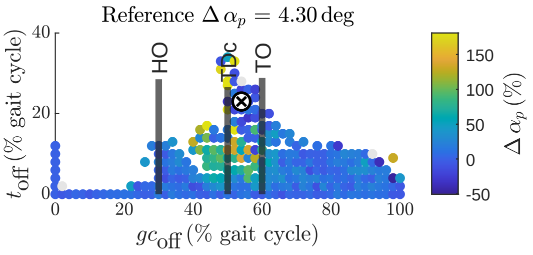

(i) Leg's momentum change angle from horizontal.

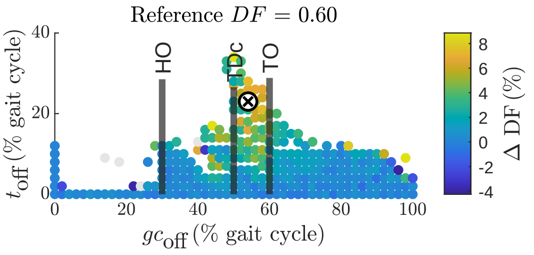

(j) Duty factor.

Figure S4: Heatmaps for turning off VAS stimulation. Outliers appear in gray and are defined as samples below 1% and above 99% percentiles of all results.

S5 Single muscle contributions (SMC) to hip, knee and ankle torques for selected trials

Figure S5 shows the individual torque contributions for all muscles around the ankle, knee, and hip joint for the fully actuated reference simulation with default control parameters from [2].

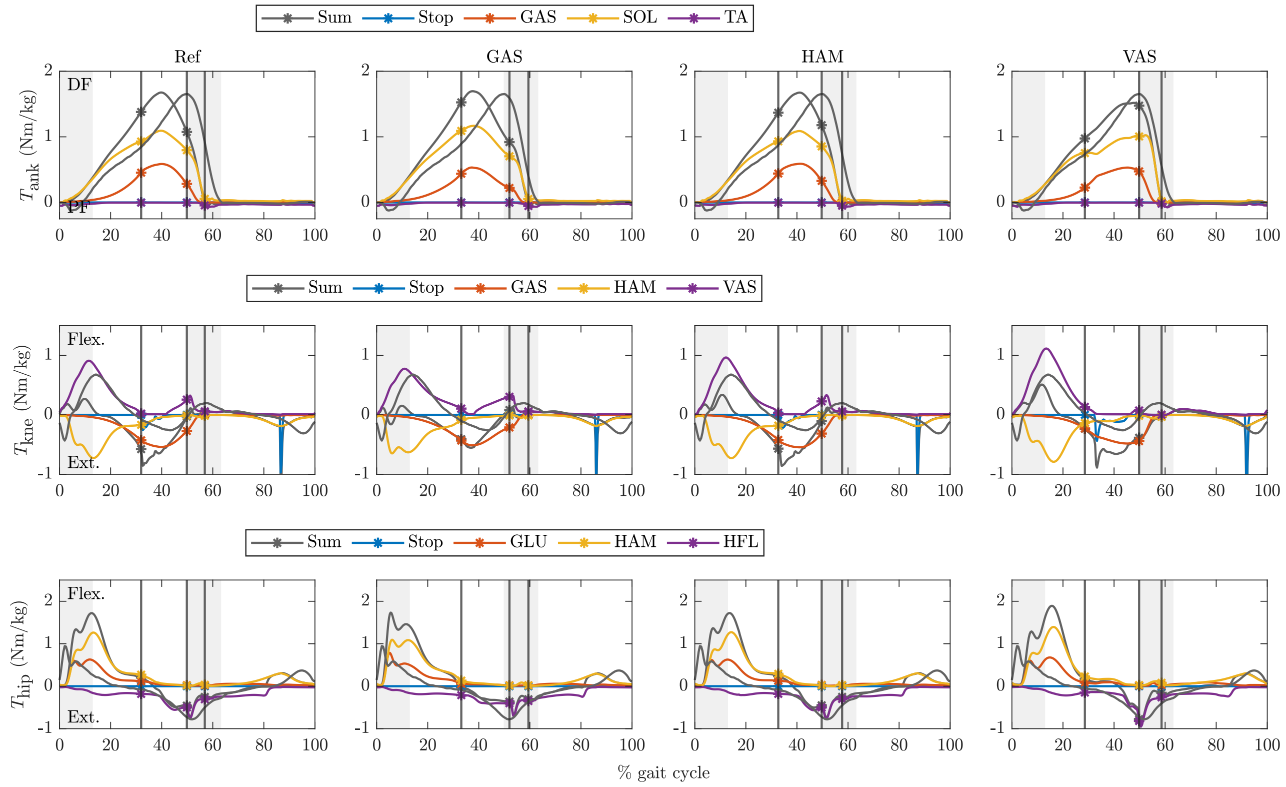

Figure S5: Muscle torque contributions for the reference simulation normalized to body weight. Gray areas indicate double support and horizontal lines show heel strike, opposite heel strike, and toe-off. Gray lines show human reference data for the total joint torque from [1]. Spring-damper joint limits implemented in the simulation with $k = 0.3\,\mathrm{Nm\,deg^{-1}}$ and $v_{max} = 1\,\mathrm{deg^{-1}}$ induce the stop-moments; see [2, Appendix IV, p.271] for details. Only the knee exhibits stop moments at about 90% of the gait cycle when the knee extends beyond $175\,\mathrm{deg}$ at the end of the swing. The hip and ankle remain within the joint limits throughout the gait cycle and have no stop-moment contributions. The ankle is spanned by gastrocnemius (GAS), soleus (SOL), and tibialis anterior (TA), the knee by vastus group (VAS), hamstrings (HAM), and GAS, and the hip by hip flexor muscles (HFL), gluteus group (GLU), and HAM. See fig. 1 for an overview of the muscles.

Ankle GAS, SOL, and TA span the ankle, the total torque increases almost linearly until the end of the stance phase and then drops rapidly to zero after push-off. SOL contributes about twice as much torque as GAS.

Knee GAS and HAM flex the knee while VAS extends the knee. HAM and GAS are biarticular muscles, VAS is monoarticular. VAS and HAM are mainly active in the first half of the stance between 0-35% gait cycle until heel-off. VAS is active to counter the impact forces during loading response and HFL to stabilize the upper body. After heel-off, VAS and HAM have no significant torque contribution, and GAS is becoming active until toe-off. The total joint torque in simulation roughly matches the shape of human data in terms of positive and negative periods. However, the knee in simulation exhibits significantly fewer flexion torques during loading response and more extension torques around midstance than human data.

Hip GLU and HAM extend and HFL flexes the hip. GLU and HAM are active at the beginning of the stance between 0-35% of the gait cycle until heel off to stabilize the upper body. HAM is activated again at the end of swing to facilitate swing leg retraction [2]. HFL shows a slightly negative torque contribution over the entire gait cycle and has its peak contribution around the opposite heel strike at 45-55% stride where swing is initiated. Especially at the beginning of stance, the total hip torques in the simulation are significantly higher than in the human data. The torques are comparable to human data around push-off and during swing.

S6 Muscle specific mechanical energy contributions in stance for hip, knee, and ankle

Table S1: Individual muscle contributions for the individual muscles to joint mechanical energy during stance for selected trials. The mechanical energy is obtained by integrating the joint power over time. ΔE^+ includes all positive areas below the power curve, ΔE^- all negative contributions. The total mechanical energy ΔE_{tot} is the sum of ΔE^+ and ΔE^-. Human reference data from [1]. Reference simulation with default control parameters from [2].

	Prop	Unit	Human	Ref	GAS	HAM	VAS
	ΔE^+	[J]	21.99	15.57	15.59	15.32	13.42
$\sum$ Ankle	ΔE^-	[J]	-13.83	-10.12	-10.01	-9.88	-11.41
	ΔE_{tot}	[J]	8.16	5.45	5.58	5.44	2.01
	ΔE^+	[J]	-	3.6	3.52	3.41	1.43
↳ GAS	ΔE^-	[J]	-	-1.84	-1.72	-1.73	-1.98
	ΔE_{tot}	[J]	-	1.76	1.8	1.68	-0.55
	ΔE^+	[J]	-	12.56	12.7	12.5	12.4
↳ SOL	ΔE^-	[J]	-	-8.04	-8.14	-7.76	-8.5
	ΔE_{tot}	[J]	-	4.52	4.56	4.74	3.9
	ΔE^+	[J]	-	0.75	0.65	0.7	0.94
↳ TA	ΔE^-	[J]	-	-1.58	-1.45	-1.68	-2.28
	ΔE_{tot}	[J]	-	-0.83	-0.79	-0.98	-1.34
	ΔE^+	[J]	6.49	2.1	2.15	2.23	10.13
$\sum$ Knee	ΔE^-	[J]	-10.72	-10.83	-5.37	-12.06	-18.35
	ΔE_{tot}	[J]	-4.23	-8.73	-3.22	-9.83	-8.22
	ΔE^+	[J]	-	5.82	4.19	5.92	6.47
↳ GAS	ΔE^-	[J]	-	-5.78	-3.72	-6	-7.92
	ΔE_{tot}	[J]	-	0.04	0.46	-0.07	-1.45
	ΔE^+	[J]	-	5.79	3.55	6.28	10.19
↳ HAM	ΔE^-	[J]	-	-5.77	-6.18	-5.46	-8.16
	ΔE_{tot}	[J]	-	0.02	-2.62	0.82	2.03
	ΔE^+	[J]	-	13.47	9.34	14.36	11.8
↳ VAS	ΔE^-	[J]	-	-5.26	-3.95	-5.69	-7.77
	ΔE_{tot}	[J]	-	8.21	5.38	8.68	4.03
	ΔE^+	[J]	10.07	26.8	20.57	28.52	35.77
$\sum$ Hip	ΔE^-	[J]	-5.33	-10.76	-11.84	-10.81	-9.9
	ΔE_{tot}	[J]	4.74	16.04	8.73	17.71	25.87
	ΔE^+	[J]	-	9.06	7.11	9.33	11
↳ GLU	ΔE^-	[J]	-	-3.29	-3.81	-3.57	-4.4
	ΔE_{tot}	[J]	-	5.77	3.3	5.76	6.59
	ΔE^+	[J]	-	20.6	15.3	21.19	27.49
↳ HAM	ΔE^-	[J]	-	-4.46	-4.88	-4.21	-5.92
	ΔE_{tot}	[J]	-	16.14	10.43	16.98	21.57
	ΔE^+	[J]	-	7.71	7.16	8.14	12.21
↳ HFL	ΔE^-	[J]	-	-13.58	-12.16	-13.17	-14.5
	ΔE_{tot}	[J]	-	-5.87	-5	-5.03	-2.29

S7 Walking Model with GRF-Visualization

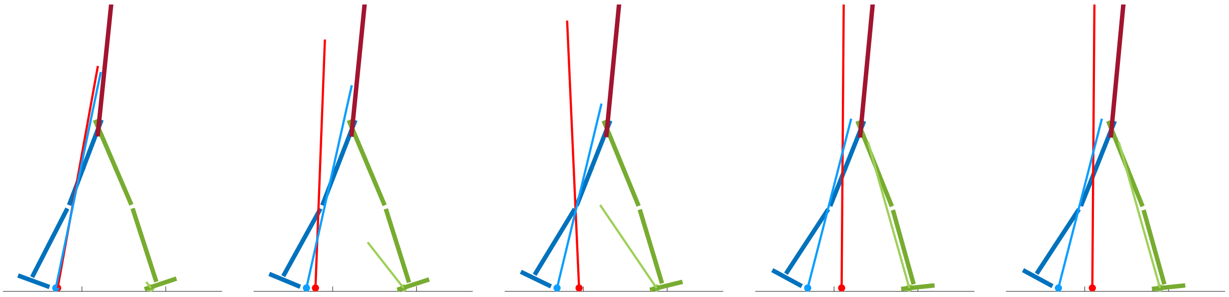

(a) 1% GC before contra-lateral leg's touch-down (TDc) | **(b)** TDc | **(c)** 11% double support (DS) after TDc | **(d)** 22% DS after TDc | **(e)** 33% DS after TDc

Figure S6: Ref - Red shows the global GRF vector, and the blue and green vectors show the individual GRF for each leg.

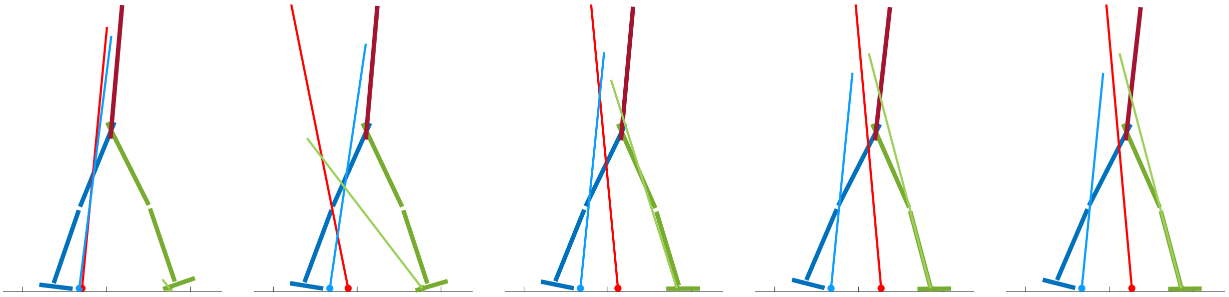

(a) 1% GC before TDc | **(b)** TDc | **(c)** 11% DS after TDc | **(d)** 22% DS after TDc | **(e)** 33% DS after TDc

Figure S7: GAS - Red shows the global GRF vector, and the blue and green vectors show the individual GRF for each leg.

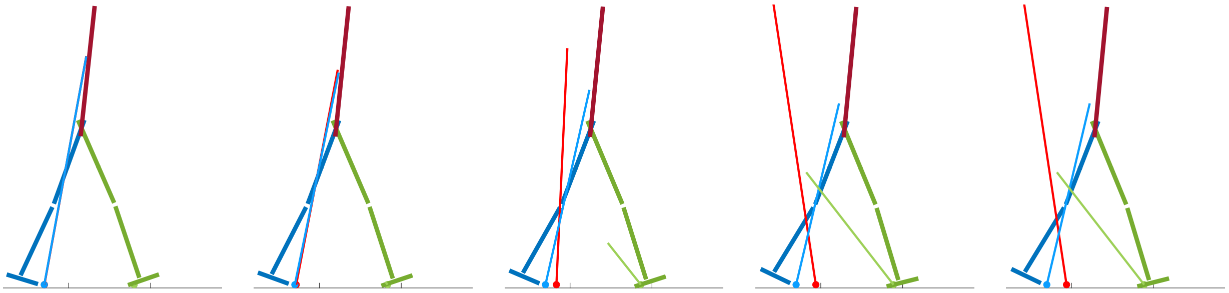

(a) 1% GC before TDc | **(b)** TDc | **(c)** 11% DS after TDc | **(d)** 22% DS after TDc | **(e)** 33% DS after TDc

Figure S8: HAM - Red shows the global GRF vector, and the blue and green vectors show the individual GRF for each leg.

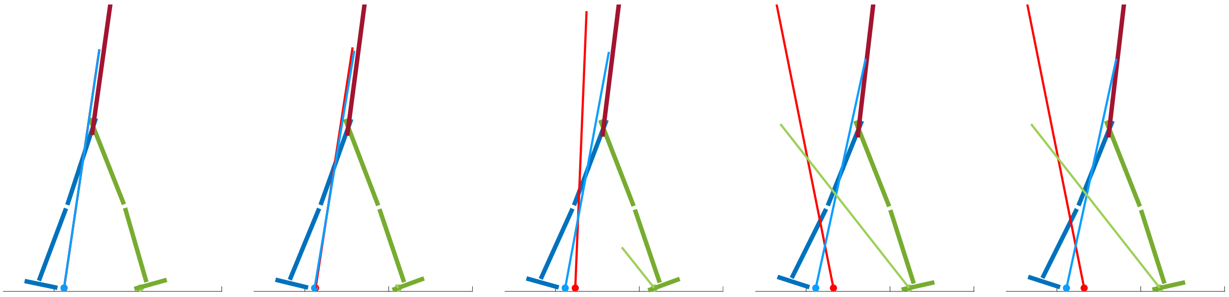

(a) 1% GC before TDc | **(b)** TDc | **(c)** 11% DS after TDc | **(d)** 22% DS after TDc | **(e)** 33% DS after TDc

Figure S9: VAS - Red shows the global GRF vector, and the blue and green vectors show the individual GRF for each leg.

S8 Gait Events and Ankle Push-off Release

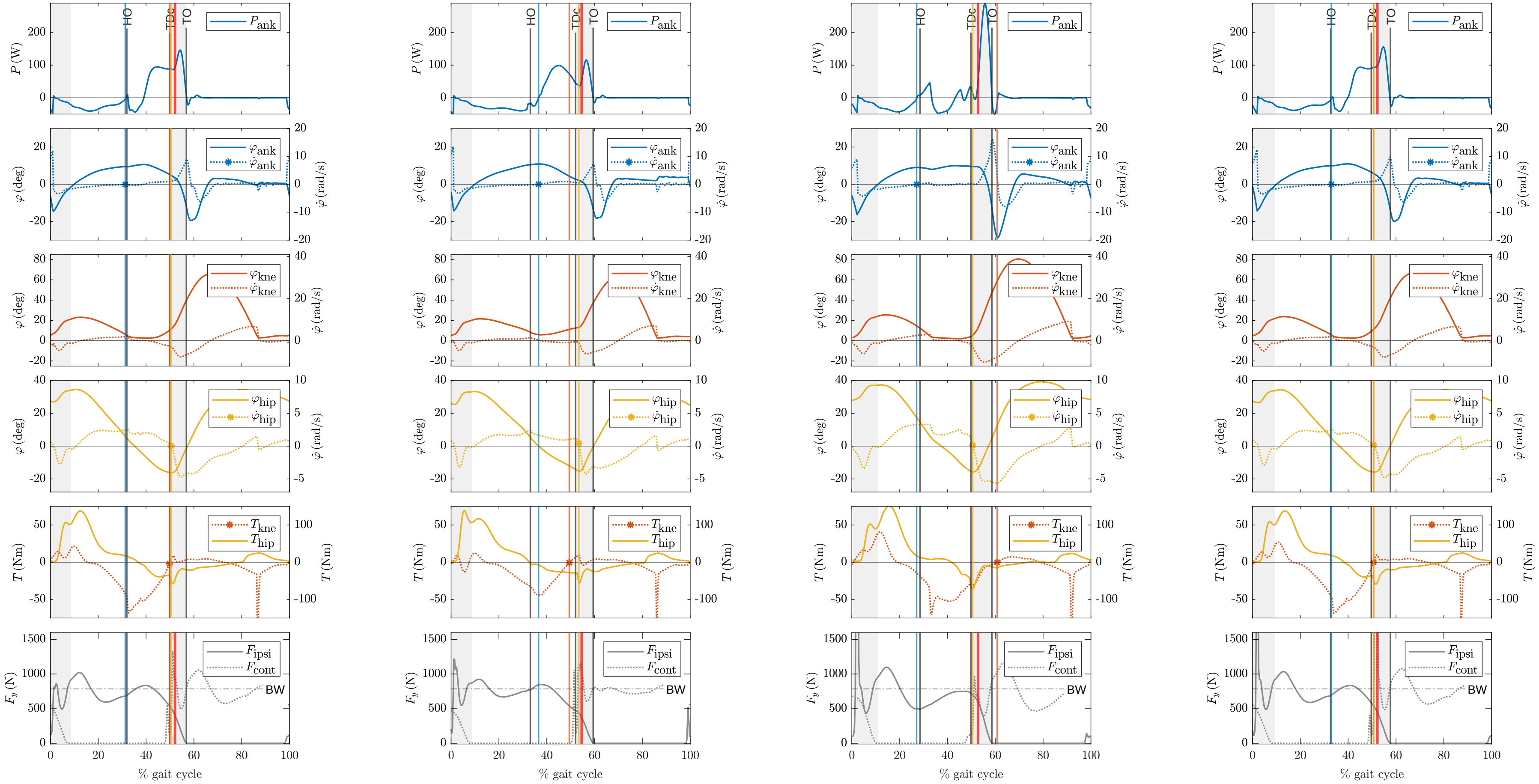

(a) Reference Simulation. **(b)** Deactivated GAS. **(c)** Deactivated VAS. **(d)** Deactivated HAM.

Figure S10: Timing of crucial gait events around ankle push-off for the different trials with deactivated muscles. Blue curves show measures related to the ankle; orange lines show measures related to the knee; yellow lines measures related to the hip and gray the vertical GRFs. The gray vertical lines mark heel-off (HO), contra-lateral leg's touch-down (TDc), and toe-off (TO), respectively. The blue vertical line indicates the zero crossing of the angular ankle joint velocity, i.e., the point of maximum ankle dorsiflexion. The yellow line marks the start of hip flexion. The orange line indicates the zero crossing of the knee torque as an indicator of the beginning of the release phase for push-off [3]. The red vertical lines in the top and bottom plot indicate the point of the last GRF snapshot shown in figs. S6 to S9.

References

[1] van der Zee, T. J., Mundinger, E. M., and Kuo, A. D. “A biomechanics dataset of healthy human walking at various speeds, step lengths and step widths”. In: *Scientific Data* 9.1 (2022), p. 704. DOI: 10.1038/s41597-022-01817-1.

[2] Geyer, H. and Herr, H. M. “A muscle-reflex model that encodes principles of legged mechanics produces human walking dynamics and muscle activities”. In: *IEEE Transactions on Neural Systems and Rehabilitation Engineering* 18.3 (2010), pp. 263–273. DOI: 10.1109/TNSRE.2010.2047592.

[3] Lipfert, S. W., Günther, M., Renjewski, D., and Seyfarth, A. “Impulsive ankle push-off powers leg swing in human walking”. In: *Journal of Experimental Biology* 217.8 (2014), pp. 1218–1228. DOI: 10.1242/jeb.097345.
